# Toasted Vine Shoots as an Alternative Enological Tool. Impact on the Sensory Profile of Tempranillo Wines during Bottle Aging

**DOI:** 10.1021/acs.jafc.2c08982

**Published:** 2023-03-24

**Authors:** R. Sánchez-Gómez, C. Cebrián-Tarancón, F. Fernández-Roldán, G. L. Alonso, M. R. Salinas

**Affiliations:** † Cátedra de Química Agrícola, E.T.S.I. Agrónomos y Montes, Universidad de Castilla-La Mancha, Avda. de España s/n, 02071 Albacete, Spain; ‡ Pago de la Jaraba, Crta, Nacional 310, km 142, 7, 02600 Villarrobledo, Spain

**Keywords:** bottle aging, enological additive, phenolic and volatile compounds, sensory analysis, vine shoots

## Abstract

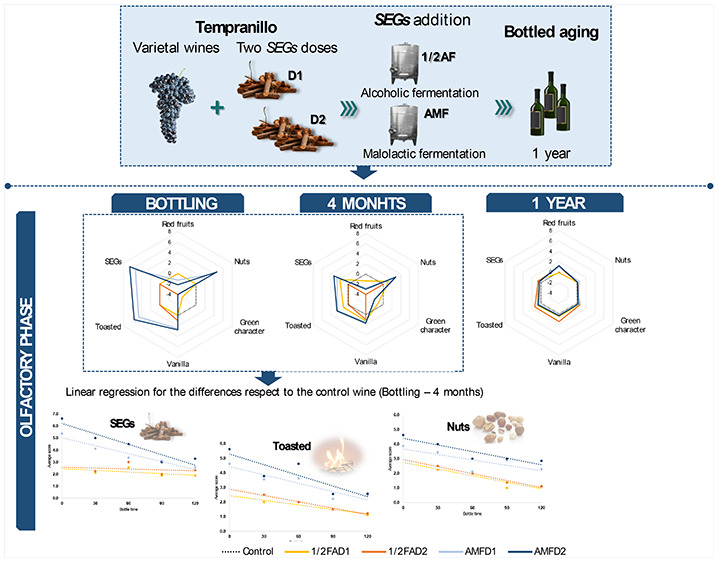

The use of toasted vine shoots (SEGs) as an enological tool is a new practice that seeks to improve wines by differentiating between them and encouraging sustainable wine production. The sensorial impact during bottle aging of wines treated with SEGs is a key factor to consider. This paper studies the influence of SEGs on Tempranillo wines treated with their own SEGs in two different doses (12 and 24 g/L) at two differences moments (during alcoholic fermentation and after malolactic fermentation) throughout 1 year of bottle aging. The results indicate that addition moment is the factor that most affects the evolution of sensorial descriptors. The greatest evolution in the wines was observed during the first 4 months, wherein improved integration of the notes related to addition of SEGs occurred. A reduction in the perception of dryness and bitterness was observed in the treated wines, therefore, SEGs could be considered accelerators to eliminate these initial sensations from wines.

## Introduction

The sustainable production of wine has been an increasingly prevalent trend in recent years and is today a reality.^1^ In the winemaking world, as in other industries, the different practices conducted in vineyards and wineries have an environmental impact. New methods of producing wine attribute greater importance to the reduction of waste or byproducts and the use of resources. Significant benefits can be achieved through the use of new noninvasive techniques and the application of natural products.

Among the principal aspects to consider for sustainable wine production are technological innovations in the fields of CO_2_ reuse, water management and saving, renewable energy, practices in enology and winemaking processes, functional biodiversity management, valorization of winemaking byproducts, and climate change adaptation.^2^ In this vein, the pruning of vine shoots represents a large annual sum of waste; for example, in Spain, that amount is approximately 2.5 million tons.^3^ In addition, the market is saturated and winemakers are continually seeking ways to differentiate their wines, always prioritizing the final quality;^4^ therefore, it is not surprising that remarkable efforts have been dedicated to valorizing vine shoots.

For some years, there has been a focus on the definition of those variables related to the preparation of the vine shoots to be used as enological additives. These vine shoots are known as SEGs (a term derived from the combination of “Shoot from vines – Enological – Granule”). Primarily, SEGs have been examined for their chemical composition,^5−8^ toxicity,^5^ repercussions on the chemical level,^9−12^ and correct dosage and moments of addition during winemaking, depending on the desired effect to be achieved.^9−12^ Few researchers have examined the scope of the sensory impact of SEGs, despite this specific impact being fundamental from the perspective of acceptance by consumers.^9^

The use of SEGs as an enological additive has been justified by their interesting chemical composition, which is achieved after temporal and thermal treatments. The vine shoots have been found to have similarities to oak wood, as both contain lignocellulosic materials. When the vine shoots are thermally treated, aromatic substances that constitute the most important group of compounds responsible for the aroma of wood arise. However, as might be expected, not all the compounds present in oak wood are present in SEGs after toasting, for example, *trans*/*cis*-whiskey lactones.^13^ Regarding tannic composition, SEGs also differ from oak in that SEGs do not have hydrolyzable tannins in their composition, only condensed tannins.^6^ These variances translate into a different sensorial perception; therefore, it is extremely important to define and use differentiated descriptors that can be associated with the character provided by the SEGs. Thus, the use of SEGs is intended, from both the sensorial and chemical viewpoints, to develop more complex wines, where the integration of all the descriptors occurs in a balanced way with no rough edges. In line with this, the time of bottle aging is decisive concerning achieving a perfect roundness of all aromas and sensations.

It has been found that a bottle period is fundamental after the contact of a wine with wood (barrels or alternative products), as wines undergo changes that entail a continuation of those processes that begin during contact with the wood, which are determined not only by the initial wine characteristics but also those of the wood and the length of the contact period.^14^ During bottle aging, a wide range of chemical reactions and wine sensory changes take place, related to color, aroma, or in-mouth properties,^15^ for example, a decrease in fresh fruit aromas and astringency.^16,17^ The pace of these changes slows down during bottle storage, but a range of chemical reactions continue, many of which are capable of generating different sensory properties, both positive and negative, that could ultimately have relevance to consumer acceptance of the product.^18^ Thus, the bottle aging period is critical in the wine value chain as, once the wine is bottled, there are no further possibilities to control any sensory deviation. Therefore, it is vital to attempt to foresee the evolution of the sensory properties of bottled wines during aging and to inform the consumer about the optimum consumption moment for reaching the desired sensory properties. Thus, the aim of this research is to study the evolution in bottles of Tempranillo wines that were in contact with different doses of SEGs of their own variety during different moments of winemaking and the effect of that contact on the sensory profile of the wines throughout 1 year.

## Materials and Methods

### Wines in Contact with SEGs

Wines used in this research were those obtained for a study by Cebrián-Tarancón et al.^10^ Briefly, a red single-variety wine (cv. Tempranillo) produced in Pago de La Jaraba cellar (Castilla-La Mancha, Spain) during the 2020 vintage, which was in contact with toasted vine shoot fragments, SEGs. Vine shoots were obtained during 2020 from Tempranillo red vinifera cultivar (T; VIVC: 12350) and transformed as an enological additive in accordance with Cebrián-Tarancón et al.^13^ The SEGs were in contact with the wine in “always full” 500 L stainless steel tanks under different conditions of dosage (D1, 12 g/L; D2, 24 g/L) and different moments of addition during the Tempranillo winemaking process. For the 1/2AF wines, SEGs were added in the middle of alcoholic fermentation and removed after 30 days of maceration; for the AMF wines, SEGs were added after malolactic fermentation and removed after 30 days of maceration. The wines were produced in duplicate; therefore, a total of eight wines were obtained, with a final volume of approximately 200 L of each.

After the SEGs were removed, the wines were bottled and aged in the same cellar, where humidity and temperature conditions were controlled at 65–75% and 15–16 °C, respectively. In addition, control wines (C), without the addition of SEGs, were obtained in duplicate. The mean values of the enological parameters of the wines, without significant differences between each treatment compared with the control, were as follows: alcoholic degree (°A) at 13.00% (v/v), TA at 4.5 g/L, and pH at 4.00. For color intensity, significant differences were observed, with the mean values recorded at 8.51 in the SEG wines and 9.52 in the control wines. These parameters were evaluated before the wines were transferred into the bottles, in accordance with OIV methods.^19^

Once bottled, the wines were sampled monthly for sensory monitoring, according to the research of Fanzone et al.^9^ up to 4 months (named M1, M2, M3, and M4). After these 4 months, the volatile and phenolic compositions were analyzed. A final sensory analysis was performed after 1 year, as 12 months is the period widely accepted as long aging in the bottle.

### Wine Analysis

#### Determination of Volatile Compounds by SBSE-GC-MS

The wine volatiles were determined according to the methodology outlined by Sánchez-Gómez et al.^20^ Their extraction was conducted by means of Stir Bar Sorptive Extraction (SBSE) (PDMS; 10 mm length; 0.5 mm film thickness) after the wine was stirred at 500 rpm for 60 min. Later, analysis was performed using an automated thermal desorption unit (TDU; Gerstel, Mülheim and der Ruhr, Germany) mounted on an Agilent 7890A gas chromatograph system (GC) coupled to a quadrupole Agilent 5975C electron ionization mass spectrometric detector (MS; Agilent Technologies, Palo Alto, CA, USA) equipped with a fused silica capillary column (BP21 stationary phase; 30 m length; 0.25 mm ID; and 0.25 μm film thickness; SGE, Ringwood, Australia). The carrier gas was helium with a constant column pressure of 20.75 psi.

The stir bars were thermally desorbed in a stream of helium carrier gas at a flow rate of 75 mL/min with the TDU programmed from 40 to 295 °C (held 5 min) at a rate of 60 °C/min in splitless desorption mode. The analytes were focused on a programmed temperature vaporizing injector (PTV) (CIS-4, Gerstel) containing a packed liner (20 mg tenax TA) held at −10 °C with cryocooling prior to injection. After desorption and focusing, the CIS-4 was programmed from −10 to 260 °C (held for 5 min) at 12 °C/min to transfer the trapped volatiles into the analytical column. The GC oven temperature was programmed to 40 °C (held for 2 min) then raised in increments: to 80 °C (5 °C/min, held for 2 min), to 130 °C (10 °C/min, held for 5 min), to 150 °C (5 °C/min, held for 5 min), and to 230 °C (10 °C/min, held for 5 min). The MS was operated in scan acquisition (27–300 *m*/*z*) with an ionization energy of 70 eV. The temperature of the MS transfer line was maintained at 230 °C. MS data acquisition was conducted in positive scan mode, although, to avoid matrix interferences, the MS quantification was performed in the single ion-monitoring mode using volatile compound characteristic *m*/*z* values. Compound identification was performed using the NIST library and confirmed by comparison with the mass spectra and retention time of volatile compound pure standards. The standards used to identify and quantify the volatile compounds (GC-MS) were purchased from Sigma-Aldrich (Steinheim, Germany). The numbers in brackets indicate the *m*/*z* used for quantification:^20^ hexanoic acid (*m*/*z* = 60), octanoic acid (*m*/*z* = 60), decanoic acid (*m*/*z* = 60), 2-phenylethanol (*m*/*z* = 91), 1-hexanol (*m*/*z* = 56), *cis*-3-hexen-1-ol (*m*/*z* = 67), benzyl alcohol (*m*/*z* = 108), nonanol (*m*/*z* = 56), benzaldehyde (*m*/*z* = 106), nonanal (*m*/*z* = 57), ethyl lactate (*m*/*z* = 45), ethyl octanoate (*m*/*z* = 101), ethyl butyrate (*m*/*z* = 88), ethyl decanoate (*m*/*z* = 101), diethyl succinate (*m*/*z* = 101), ethyl vanillate (*m*/*z* = 151), ethyl hexanoate (*m*/*z* = 101), ethyl cinnamate (*m*/*z* = 131), ethyl acetate (*m*/*z* = 43), isoamyl acetate (*m*/*z* = 43), 2-phenylethyl acetate (*m*/*z* = 104), hexyl acetate (*m*/*z* = 43), β-ionol (*m*/*z* = 205), β-damascenone (*m*/*z* = 121), α-ionone (*m*/*z* = 121), β-ionone (*m*/*z* = 177), geraniol (*m*/*z* = 69), citronellol (*m*/*z* = 69), farnesol (*m*/*z* = 69), linalool (*m*/*z* = 71), nerolidol (*m*/*z* = 69), guaiacol (*m*/*z* = 109), eugenol (*m*/*z* = 164), and vanillin (*m*/*z* = 151). 3-Methyl-1-pentanol was used as the internal standard. Quantification was based on calibration curves of the respective standards at five different concentrations (*R*^2^ = 0.95–0.97). All analyses were made in triplicate. The linear range, detection and quantification limits (LOD and LOQ), %RSD, and recovery, as well as the CAS number, are summarized in Table S1 (supplementary data).

#### Determination of Low Molecular Weight Phenolic Compounds by HPLC-DAD

The wines’ low molecular weight phenolic compounds were determined according to the work of Cebrián-Tarancón et al.^11^ A volume of 20 μL of wine was injected into an Agilent 1200 HPLC chromatograph (Palo Alto, California, USA), which was equipped with a Diode Array Detector (DAD; Agilent G1315D) coupled to an Agilent ChemStation (version B.03.01) data processing station. Separation was performed in a reverse phase ACE C18-PFP (4.6 mm × 150 mm; 3 μm particle size) and a precolumn ACE Excel HPLC Precolumn Filter 1PK (0.5 μm particle size) at 30 °C. The HPLC proportion of solvents used was water/formic acid/acetonitrile (97.5:1.5:1 v/v/v) for solvent A and acetonitrile/formic acid/solvent A (78.5:1.5:20 v/v/v) for solvent B. The elution gradient was set up for solvent B as 0 min, 5%; 8.40 min, 5%; 12.50 min, 10%; 19 min, 15%; 29 min, 16%; 30 min, 16.5%; 34.80 min, 18%; 37.20 min, 32%; 42 min, 62%; 52 min, 90%; 54 min, 100%; 56 min, 100%; 60 min, 5%; and 65 min, 5%. All compound detection was completed using the DAD in comparison with the corresponding UV–vis spectra and retention time of the compounds’ pure standards. Quantification and identification of the analyzed compounds was outlined in more detail in the work of Cebrián-Tarancón et al.^11^ Briefly, the wavelengths were (+)-catechin, (−)-epicatechin, procyanidin B2, gallic acid, protocatechuic acid, syringic acid, and 4-hidroxybenzoic acid at 280 nm; ellagic acid and vanillic acid at 256 nm; *trans*-caffeic acid and, *trans*-caftaric acid at 324 nm; *trans*-coumaric acid, *trans*-coutaric acid, and *trans*-resveratrol at 308 nm; delphinidin 3-*O*-glucoside, cyanidin 3-*O*-glucoside, petunidin 3-*O*-glucoside, peonidin 3-*O*-glucoside, malvidin 3-*O*-glucoside, malvidin 3-(6′-acetyl)-glucoside, malvidin 3-(6′-caffeoyl)-glucoside, cyanidin 3-(6′-*p*-coumaroyl)-glucoside, petunidin 3-(6′-*p*-coumaroyl)-glucoside, and malvidin 3-(6′-*p*-coumaroyl)-glucoside at 520 nm; and myricetin 3-*O*-galactoside, myricetin 3-*O*-glucuronide, myricetin 3-*O*-glucoside, laricitrin 3-*O*-glucuronide/galactoside, syringetin 3-*O*-glucoside, myricetin, and quercetin at 365 nm. The anthocyanins analyzed were quantified in terms of malvidin 3-*O*-glucoside, flavanols in terms of quercetin 3-*O*-glucoside, *trans*-caftaric in terms of *trans*-caffeic acid, and *trans*-coutaric in terms of *trans*-coumaric. Quantification was based on the calibration curves of the respective standards (Sigma-Aldrich, Steinheim, Germany) at five different concentrations achieved using UV–vis signal (*R*^2^ = 0.92–0.99). All analyses were completed in duplicate. The linear range, detection and quantification limits (LOD and LOQ), %RSD, and recovery, as well as the CAS number, are summarized in Table S2 (supplementary data).

#### Sensory Analysis of the Wines

A group of nine panelists (three females and six males aged between 25 and 65 years old) with previous experience in the preparation and maceration of SEGs and therefore trained in recognizing SEGs’ aromatic characteristics, participated in the tasting. This training consisted of offering the panelists synthetic wines with five doses of toasted SEGs, which were used as references to establish their intensity on a five-point scale (1 = not intense; 5 = very intense). Six sensorial analyses were conducted, corresponding to the following moments: bottling, M1–M4, and after 1 year. In each of these sensorial analyses, the four wines from the treatments (1/2AFD1, 1/2AFD2, AMFD1, and AMFD2) were evaluated and compared to the control (C). Some information was given to the panelists about the origin of the samples. The order of the wines sampled, unknown to the panelists, was 1/2AFD1, 1/2AFD2, AMFD1, and AMFD2. All tastings were performed in a wine tasting room located in Pago de la Jaraba cellar (Villarrobledo, Spain), with the intention of minimizing any external influence. The room was air conditioned (21 °C) with a round table and optimal conditions to facilitate the tasters’ task of the sensory evaluation of wine.

As the aim was to evaluate the treatment effect, two bottles from the same treatment (one per sensorial analysis) were mixed prior to each tasting session. Thus, in each tasting moment, five wines were analyzed. Considering that SEGs can give wine aromas that have not yet been defined, an adapted tasting evaluation sheet that included new descriptors was created. The odor attributes to evaluate were determined by consensus after the panel discussed reducing the number of descriptors in one dedicated session before the measure sessions. In each sampling session, the wines were assessed by the panelists with consideration of 18 descriptors, which were grouped by visual phase (purple, garnet, and red), olfactory phase (red fruits, nuts, green character, vanilla, toasted, and SEGs), taste phase (red fruits, nuts, green, vanilla, toasted, and SEGs), and tannins (dryness, silkiness, and bitterness).

All evaluations were conducted from 10:30 am to 1:00 pm. Constant volumes of 30 mL of each wine were evaluated in wine taster glasses at 21 °C, in accordance with ISO 3591 (1977). Prior to the individual evaluation of each treatment, the control wine was jointly analyzed to establish a consensual assessment among all the tasters. Panellists were provided with unsalted crackers and water and were asked to rinse their mouths and wait for a minimum of 2 min between evaluation of each sample. After that, the panelists smelled and tasted the different wines, noted the specific descriptors perceived, and rated the intensity of each sensory descriptor on an 11-point scale, where 0 indicated that the descriptor was not perceived (absence) and values from 1 to 10 indicated very low to maximum. All the sensory evaluations were completed under Spanish Standardisation Rules (6658:2017, 1997).

#### Data Analysis

Statistical analyses were performed using the Statgraphics Centurion statistical program (version 19.4.02; StatPoint, Inc., The Plains, VA, USA). Multivariate analysis of variance (MANOVA) was conducted to compare sensory analysis descriptors for all the wine treatments studied (1/2AFD1, 1/2AFD2, AMFD1, and AMFD2), with particular attention to the dose (D1, 12 g/L; D2, 24 g/L) and the different moment of addition during the Tempranillo winemaking process (1/2AF, SEGs added in the middle of alcoholic fermentation; AMF, SEGs added after malolactic fermentation).

The descriptive analysis results regarding wine composition (volatile and phenolic compounds) were examined using one-way analysis of variance (ANOVA) at a 95% probability level, in accordance with Fisher’s least significant difference (LSD) test, to determine the differences between wines. A linear regression fitting was considered to establish the evolution of sensorial descriptors related to the SEGs’ impact in the first 4 months of bottle aging. A principal component analysis (PCA) was performed with the purpose of obtaining an overall view of the influence of SEG treatments in bottle aging.

## Results and Discussion

Previous research related to the use of vine shoots as enological additives has focused mainly on the effect on the chemical composition of wines.^9−11^ Few works have focused on the impact of vine shoots as enological additives at a sensory level and the evolution throughout short bottle aging.^9^ It has been found that the SEGs’ sensory impact is highly relevant concerning acceptance by the consumer, differentiation from control wines, and intensity and persistence. The contribution of the studied factors (dose and moment of addition of SEGs during winemaking) to the sensorial descriptors is outlined in Table 1. The chemical composition is summarized in Tables 2–5, where the content of volatile and phenolic compounds of each of the studied treatments (1/2AFD1, 1/2AFD2, AMFD1, and AMFD2) and the statistical information in response to two one-way ANOVA tests is depicted. For each treated wine, significant differences with respect to the control wine are indicated according to *p <0.05, **p <0.01, and ***p <0.001.

**Table 1:** *F*-Values from the Multivariate Analysis of Variance (MANOVA) of Differences with Respect to Control Wine for Sensorial Descriptors Attending to Dose and Moment Factors at Bottling, 4 Months, and 1 Year^a^

descriptor	dose (D)	moment (M)	D × M
Visual			
purple	0.08	20.93***	0.08
garnet	0.10	0.95	0.00
red	0.00	24.59***	0.00
Olfactory			
red fruits	15.20*	3.18	4.60*
nuts	1.14	85.02***	1.14
herbaceous	0.81	7.29**	0.81
vanilla	1.26	51.91***	1.79
toasted	6.51*	102.11***	0.43
SEGs	1.60	55.69***	4.81*
Taste			
red fruits	11.16**	0.49	9.67**
nuts	2.59	101.03***	0.02
herbaceous	0.00	0.00	0.00
vanilla	1.26	51.91***	1.79
toasted	6.51*	102.11***	0.43
SEGs	1.60	55.69***	4.81*
Tannin			
dryness	1.62	5.16*	1.43
silkiness	9.07**	26.63***	0.75
bitterness	1.95	0.62	0.15

a Significant differences according to Fisher’s LSD test are indicated as *p < 0.05, **p < 0.01 or ***p < 0.001.

**Table 2:** Volatile Compound Concentration (μg/L) of Wines Elaborated in Contact with SEGs Added at Two Different Moments and Bottling for 4 Months^a^

	C	1/2AFD1	1/2AFD2	AMFD1	AMFD2
Acids					
hexanoic acid	1816.82 ± 151.33	2007.28 ± 210.49	2850.68 ± 504.34*	2220.08 ± 267.56	2312.04 ± 239.59*
octanoic acid	1645.96 ± 122.54	1678.67 ± 136.27	2044.86 ± 174.17*	1438.93 ± 160.30	1364.94 ± 76.56*
decanoic acid	257.30 ± 8.67	233.50 ± 18.27	249.38 ± 29.01	100.47 ± 13.40***	74.35 ± 4.12***
total acids	3720.08 ± 274.50	3919.45 ± 328.01	5144.92 ± 690.20*	3759.48 ± 424.58	3751.34 ± 320.26
Alcohols					
2-phenylethanol	4280.18 ± 60.29	4196.77 ± 218.99	3447.54 ± 297.46**	3141.88 ± 380.81**	3856.50 ± 183.95*
1-hexanol	988.40 ± 61.65	1036.54 ± 28.54	1584.91 ± 12.66***	1549.95 ± 180.54**	1298.79 ± 78.63**
*cis-*3-hexen-1-ol	185.29 ± 1.29	183.79 ± 1.19	184.88 ± 2.32	186.63 ± 6.57	201.73 ± 3.50**
benzyl alcohol	164.99 ± 11.74	98.90 ± 5.68***	112.15 ± 6.02**	202.25 ± 18.11*	243.78 ± 11.59**
nonanol	4.26 ± 0.37	3.20 ± 0.33*	3.48 ± 0.34	4.68 ± 0.32	4.07 ± 0.34
total alcohols	5623.12 ± 127.85	5519.22 ± 225.66	5332.95 ± 315.57	5085.39 ± 584.15	5604.86 ± 278.02
Aldehydes					
benzaldehyde	3.86 ± 0.41	3.24 ± 0.28	3.10 ± 0.48	3.57 ± 0.13	4.44 ± 0.05
nonanal	2.69 ± 0.04	2.41 ± 0.35	2.76 ± 0.17	2.92 ± 0.61	3.02 ± 0.09**
total aldehydes	6.54 ± 0.45	5.65 ± 0.55	5.86 ± 0.65	6.49 ± 0.72	7.46 ± 0.13*
Esters					
Ethyl esters					
ethyl lactate	202301.16 ± 24399.05	160364.86 ± 10192.87	138896.55 ± 13082.64*	435343.16 ± 57463.56**	98524.31 ± 3651.29**
ethyl octanoate	3214.12 ± 71.60	2796.55 ± 258.22	3279.99 ± 389.22	3302.54 ± 277.20	3349.90 ± 113.46
ethyl butyrate	382.53 ± 36.15	422.07 ± 6.55	420.39 ± 25.62	335.36 ± 38.22	278.59 ± 10.72**
ethyl decanoate	86.70 ± 0.40	36.32 ± 5.33***	46.83 ± 5.08***	20.00 ± 0.76***	12.95 ± 0.36***
diethyl succinate	5084.78 ± 578.95	1190.13 ± 5.62***	1634.72 ± 27.46***	4995.00 ± 526.24	3977.11 ± 56.05*
ethyl vanillate	248.80 ± 24.63	203.35 ± 19.86	190.14 ± 29.20	304.73 ± 34.02	191.98 ± 9.15*
ethyl hexanoate	37.57 ± 1.11	35.77 ± 1.91	47.54 ± 5.43*	49.62 ± 4.23**	49.99 ± 2.54**
ethyl cinnamate	0.19 ± 0.02	0.21 ± 0.03	0.18 ± 0.01	0.30 ± 0.14	0.48 ± 0.04***
Acetates					
ethyl acetate	32131.28 ± 1868.09	34899.56 ± 1600.70	33316.57 ± 2166.11	29364.72 ± 1881.34	19607.36 ± 2158.94**
isoamyl acetate	430.88 ± 37.12	428.64 ± 8.24	381.29 ± 17.52	443.32 ± 47.81	287.60 ± 3.32**
2-phenyl ethyl acetate	14.03 ± 1.50	15.45 ± 1.70	11.29 ± 1.63	10.69 ± 1.06*	13.05 ± 0.80
hexyl acetate	1.55 ± 0.04	1.42 ± 0.09	1.71 ± 0.11	1.75 ± 0.20	1.37 ± 0.09*
total esters	243933.59 ± 26861.15	200394.34 ± 11536.34	178227.20 ± 14850.88*	474171.17 ± 59193.43**	126294.68 ± 6006.76**
Norisoprenoids					
β-ionol	38.68 ± 0.53	24.23 ± 0.86***	20.76 ± 1.28***	20.01 ± 0.76***	20.61 ± 0.02***
β-damascenone	7.40 ± 0.13	7.60 ± 0.35	7.95 ± 0.28*	7.16 ± 0.21	7.88 ± 0.13*
α-ionone	0.23 ± 0.01	0.22 ± 0.01***	0.22 ± 0.02***	0.23 ± 0.01	0.23 ± 0.01
β-ionone	0.27 ± 0.00	0.28 ± 0.01	0.29 ± 0.01**	0.31 ± 0.01**	0.35 ± 0.00***
total norisoprenoids	46.58 ± 0.48	32.33 ± 1.20***	29.21 ± 1.29***	27.71 ± 0.94***	29.06 ± 0.15***
Terpenes					
geraniol	14.57 ± 0.99	7.88 ± 0.39***	9.20 ± 0.25***	14.43 ± 1.33	13.48 ± 0.40
citronellol	7.65 ± 0.41	6.98 ± 0.51	6.05 ± 1.07	6.19 ± 1.17	5.37 ± 1.00***
farnesol	7.64 ± 0.06	3.98 ± 1.78*	4.58 ± 0.24***	6.09 ± 0.36**	2.87 ± 0.02***
linalool	3.42 ± 0.12	3.02 ± 0.10*	3.07 ± 0.14*	3.02 ± 0.04**	3.12 ± 0.04*
nerolidol	2.07 ± 0.05	1.51 ± 0.11**	1.51 ± 0.05***	1.52 ± 0.06***	1.31 ± 0.02***
total terpenes	35.35 ± 1.44	23.37 ± 2.87**	24.42 ± 0.74***	31.26 ± 2.08*	26.16 ± 1.36**
Volatile Phenols					
guaiacol	17.37 ± 2.31	16.63 ± 1.01	15.41 ± 2.48	19.27 ± 1.58	18.04 ± 1.07
eugenol	10.35 ± 0.52	10.23 ± 0.57	11.39 ± 0.80	15.39 ± 0.71***	16.30 ± 0.13***
vanillin	2.05 ± 0.70	1.51 ± 0.85	2.06 ± 1.10	2.53 ± 0.87	2.79 ± 0.02
total volatile phenols	29.77 ± 3.03	28.07 ± 2.10	28.86 ± 4.37	37.19 ± 1.91*	37.13 ± 1.18*
Total Volatile Compounds	253395.03 ± 27111.11	209922.73 ± 11366.73	188793.42 ± 13921.42*	483118.69 ± 59909.41**	135750.69 ± 6607.85**

a ANOVA results of differences respect to control wine. C, control wine untreated with SEGs; 1/2AFD1, 12 g/L of SEGs added in the middle of alcoholic fermentation; 1/2AFD2, 24 g/L of SEGs added in the middle of alcoholic fermentation; AMFD1, 12 g/L of SEGs added after malolactic fermentation; AMFD2, 24 g/L of SEGs added after malolactic fermentation. The mean values (*n* = 4) are shown with their standard deviation. For each compound and each treated wine, significant differences according to Fisher’s LSD test are indicated respect to control wine in each wine column (*p < 0.05; **p < 0.01. ***p <0.001).

**Table 3:** Low Molecular Weight Phenolic Compounds (mg/L) of Wines Elaborated in Contact with SEGs Added and Bottling for 4 Months^a^

	C	1/2AFD1	1/2AFD2	AMFD1	AMFD2
Flavanols					
(+)-catechin	77.32 ± 4.38	64.87 ± 0.85	61.55 ± 1.03*	59.35 ± 2.02*	65.69 ± 1.43
(−)-epicatechin	1114.31 ± 68.80	1086.44 ± 75.67	805.52 ± 44.06*	756.60 ± 34.45*	850.48 ± 75.78
procyanidin B2	13.45 ± 2.32	12.34 ± 0.24	12.89 ± 0.22	15.72 ± 2.35	13.06 ± 0.05
total flavanols	1205.07 ± 62.10	1163.66 ± 74.57	879.95 ± 42.81*	831.66 ± 38.83*	929.22 ± 77.16
Phenolic Acids					
ellagic acid	56.82 ± 3.82	52.25 ± 1.34	47.62 ± 0.85	46.63 ± 0.79	49.84 ± 0.42
gallic acid	54.78 ± 0.13	44.79 ± 0.19***	41.50 ± 1.46**	24.07 ± 0.13***	12.46 ± 0.08***
protocatechuic acid	4.53 ± 0.99	3.72 ± 0.06	2.96 ± 0.28	b	b
syringic acid	3.41 ± 0.10	3.33 ± 0.14	3.83 ± 0.31	6.62 ± 0.22**	4.84 ± 0.00**
*t*-caffeic acid	b	b	b	b	b
*t*-caftaric acid^c^	22.79 ± 0.49	18.12 ± 0.06**	14.05 ± 0.08**	7.33 ± 0.14***	13.28 ± 0.09**
*t-*coumaric acid	b	b	b	b	b
*t*-coutaric acid^d^	7.48 ± 0.07	6.64 ± 1.32	4.98 ± 0.13**	2.06 ± 0.04***	3.18 ± 0.02***
4-hydroxybenzoic acid	1.28 ± 0.89	1.20 ± 0.35	1.74 ± 0.09	3.46 ± 0.02	1.50 ± 0.37
vanillic acid	1.95 ± 0.03	1.58 ± 0.11*	1.54 ± 0.05**	2.47 ± 0.17	2.37 ± 1.86
total phenolic acids	153.02 ± 4.20	131.65 ± 0.54*	118.21 ± 1.65**	92.62 ± 1.07**	87.45 ± 1.73**
Stilbenes					
*t*-resveratrol	b	1.43 ± 0.06***	1.35 ± 0.06***	1.76 ± 0.03***	2.11 ± 0.10***
total stilbenes	b	1.43 ± 0.06***	1.35 ± 0.06***	1.76 ± 0.03***	2.11 ± 0.10***
Anthocyanins					
delphinidin 3-*O*-glucoside^e^	18.12 ± 0.30	18.32 ± 0.71	17.69 ± 0.49	18.74 ± 0.28	16.25 ± 0.29*
cyanidin 3-*O*-glucoside^e^	b	b	b	b	b
petunidin 3-*O*-glucoside^e^	32.24 ± 0.68	35.07 ± 0.25*	35.35 ± 0.26*	33.78 ± 0.60	31.12 ± 0.33
peonidin 3-*O*-glucoside^e^	b	b	b	b	b
malvidin 3-*O*-glucoside	143.55 ± 3.02	159.43 ± 4.01*	168.79 ± 3.56*	152.82 ± 1.52	171.74 ± 2.73*
malvidin 3-(6′-acetyl)-glucoside	13.60 ± 0.66	14.71 ± 0.05	16.12 ± 0.00*	13.48 ± 0.42	16.54 ± 0.35*
malvidin 3-(6′*-t*-caffeoyl)-glucoside^e^	7.80 ± 0.32	7.96 ± 0.12	8.03 ± 0.48	7.24 ± 0.23	6.02 ± 0.03*
cyanidin 3-(6′-*p*-coumaroyl)-glucoside^e^	b	b	b	b	b
petunidin 3-(6′-*p*-coumaroyl)-glucoside^e^	6.76 ± 0.17	6.79 ± 0.01	7.16 ± 0.13	6.40 ± 0.05	6.03 ± 0.06*
malvidin 3-(6′-*p*-coumaroyl)-glucoside^e^	27.28 ± 1.72	28.52 ± 0.01	32.14 ± 0.68	25.69 ± 0.20	21.06 ± 0.06*
total anthocyanins	249.35 ± 4.94	270.80 ± 5.15	285.27 ± 4.22*	258.15 ± 0.76	268.76 ± 3.21*
Flavonols					
myricetin 3-*O*-galactoside^f^	b	b	b	b	b
myricetin 3-*O*-glucuronide + myricetin 3-*O*-glucoside^f^	2.43 ± 0.01	2.04 ± 0.05**	1.85 ± 0.02***	1.94 ± 0.03**	1.91 ± 0.03**
laricitrin 3-*O*-glucuronide/galactoside^f^	b	b	b	b	b
syringetin 3-*O*-glucoside^f^	2.45 ± 0.08	2.10 ± 0.02*	2.01 ± 0.04*	2.17 ± 0.03*	1.75 ± 0.05**
myricetin^f^	5.24 ± 0.40	3.06 ± 0.11*	2.37 ± 0.12*	2.97 ± 0.10*	2.04 ± 0.04**
quercetin^f^	4.24 ± 0.09	2.85 ± 0.05**	2.16 ± 0.03**	2.47 ± 0.05**	1.66 ± 0.03***
total flavonols	14.36 ± 0.21	10.05 ± 0.01**	8.39 ± 0.09***	9.55 ± 0.04**	7.36 ± 0.07***
Total Phenolic Compounds	1621.79 ± 71.03	1577.59 ± 80.19	1293.17 ± 45.53*	1193.75 ± 40.59*	1294.91 ± 82.07

a ANOVA results of differences respect to control wine. C, control wine untreated with SEGs; 1/2AFD1, 12 g/L of SEGs added in the middle of alcoholic fermentation; 1/2AFD2, 24 g/L of SEGs added in the middle of alcoholic fermentation; AMFD1, 12 g/L of SEGs added after malolactic fermentation; AMFD2, 24 g/L of SEGs added after malolactic fermentation. The mean values (*n* = 4) are shown with their standard deviation. For each compound and each treated wine, significant differences according to Fisher’s LSD test are indicated with respect to control wine in each wine column (*p < 0.05; **p < 0.01; ***p < 0.001).

b Not detected.

c *t*-Caftaric acid was quantified in terms of *t*-caffeic acid.

d *t*-Coutaric acid was quantified in terms of *t*-coutaric acid.

e Anthocyanins were quantified in terms of malvidin 3-*O*-glucoside.

f Flavonols were quantified in terms of quercetin 3-*O*-glucoside.

**Table 4:** Volatile Compound Concentration (μg/L) of Wines Elaborated in Contact with SEGs Added at Two Different Moments and Bottling for 1 Year^a^

	C	1/2AFD1	1/2AFD2	AMFD1	AMFD2
Acids					
hexanoic acid	1452.10 ± 105.69	1200.60 ± 223.41	2462.21 ± 214.58**	2456.66 ± 428.82*	3139.39 ± 123.72***
octanoic acid	1599.44 ± 78.48	1389.06 ± 200.61	2180.19 ± 94.55**	2527.12 ± 141.39***	1683.79 ± 45.44
decanoic acid	299.74 ± 15.07	251.45 ± 52.45	408.19 ± 20.41**	168.99 ± 5.16***	65.74 ± 2.29***
total acids	3351.28 ± 192.04	2841.11 ± 471.55	5050.59 ± 329.53**	5152.77 ± 565.04**	4888.92 ± 144.81***
Alcohols					
2-phenylethanol	9818.32 ± 772.55	8330.68 ± 633.28	7491.89 ± 191.79**	10801.77 ± 344.76	8909.72 ± 225.65
1-hexanol	993.47 ± 89.52	862.85 ± 135.57	1634.07 ± 108.77**	1813.91 ± 6.94***	1406.61 ± 41.29**
*cis-*3-hexen-1-ol	190.25 ± 7.89	182.18 ± 9.81	193.43 ± 6.09	203.88 ± 9.39	222.57 ± 3.43**
benzyl alcohol	244.91 ± 11.32	122.44 ± 14.60***	130.89 ± 4.06***	510.58 ± 24.08***	381.70 ± 6.72***
nonanol	3.77 ± 0.37	2.96 ± 0.54	3.66 ± 0.18	7.45 ± 0.53***	4.10 ± 0.07
total alcohols	11250.71 ± 880.42	9501.10 ± 754.52	9453.95 ± 310.90*	13337.58 ± 366.92*	10924.71 ± 267.91
Aldehydes					
benzaldehyde	2.09 ± 0.14	1.48 ± 0.92	3.33 ± 0.03***	5.46 ± 0.25***	1.77 ± 1.08
nonanal	1.87 ± 0.04	1.81 ± 0.27	2.00 ± 0.05*	2.76 ± 0.17***	1.82 ± 0.07
total aldehydes	3.96 ± 0.18	3.30 ± 0.75	5.34 ± 0.02***	8.22 ± 0.43***	3.59 ± 1.13
Esters					
Ethyl esters					
ethyl lactate	158285.97 ± 10825.35	79550.06 ± 8157.89***	67260.38 ± 219.67***	176582.91 ± 14237.36	307070.28 ± 9487.71***
ethyl octanoate	3487.48 ± 105.16	3441.44 ± 744.77	5432.65 ± 355.79***	8187.63 ± 703.26***	4104.86 ± 46.30***
ethyl butyrate	282.35 ± 19.46	225.46 ± 35.31	220.93 ± 0.25**	105.34 ± 8.14***	263.17 ± 5.62
ethyl decanoate	41.12 ± 1.57	38.17 ± 10.08	65.02 ± 6.95**	43.27 ± 8.25	11.47 ± 0.22***
diethyl succinate	4157.49 ± 208.81	1886.17 ± 85.94***	1763.13 ± 11.90***	4103.74 ± 411.95	3943.63 ± 51.04
ethyl vanillate	625.33 ± 22.39	416.94 ± 91.40*	468.56 ± 7.32***	1156.84 ± 58.08***	434.78 ± 16.91***
ethyl hexanoate	44.30 ± 1.96	41.31 ± 6.94	70.82 ± 3.42***	95.52 ± 4.67***	70.53 ± 0.61***
ethyl cinnamate	0.29 ± 0.02	0.46 ± 0.11	0.41 ± 0.03**	1.03 ± 0.04***	0.94 ± 0.04***
Acetates					
ethyl acetate	27926.17 ± 2501.86	18687.10 ± 2490.19*	17014.67 ± 1880.30**	10186.29 ± 1648.19***	29357.58 ± 719.66
isoamyl acetate	315.27 ± 16.43	227.83 ± 27.29**	199.63 ± 1.54***	301.22 ± 26.92	626.78 ± 4.85***
2-phenyl ethyl acetate	16.81 ± 0.94	16.46 ± 2.40	14.59 ± 0.56*	29.83 ± 1.11***	21.91 ± 0.81**
hexyl acetate	1.69 ± 0.04	1.41 ± 0.30	2.76 ± 0.32**	5.59 ± 0.24***	3.85 ± 0.08***
total esters	195184.26 ± 13308.33	104532.82 ± 10102.30***	92513.55 ± 2023.88***	200799.22 ± 17108.14	345909.79 ± 9892.82***
Norisoprenoids					
β-ionol	34.31 ± 1.05	24.66 ± 1.74**	22.11 ± 0.17***	21.82 ± 0.27***	20.45 ± 0.10***
β-damascenone	7.29 ± 0.13	7.35 ± 0.44	8.25 ± 0.20**	9.84 ± 0.20***	9.36 ± 0.09***
α-ionone	0.23 ± 0.01	0.23 ± 0.01	0.25 ± 0.01**	0.24 ± 0.01**	0.23 ± 0.01
β-ionone	0.26 ± 0.01	0.28 ± 0.01	0.32 ± 0.01***	0.38 ± 0.01***	0.39 ± 0.01***
total norisoprenoids	42.10 ± 1.17	32.53 ± 2.18**	30.92 ± 0.39***	32.29 ± 0.07***	30.43 ± 0.18***
Terpenes					
geraniol	13.18 ± 1.04	6.40 ± 1.09**	8.61 ± 0.99**	22.33 ± 1.43***	13.54 ± 0.60
citronellol	5.84 ± 0.43	5.84 ± 0.79	6.51 ± 0.10	9.89 ± 0.42**	4.91 ± 0.08*
farnesol	11.95 ± 0.60	6.95 ± 1.89*	7.78 ± 0.38***	16.93 ± 0.49***	9.36 ± 0.81*
linalool	4.35 ± 0.15	3.34 ± 0.24**	3.72 ± 0.06**	5.73 ± 0.19***	4.64 ± 0.09*
nerolidol	1.78 ± 0.09	1.46 ± 0.23	1.63 ± 0.06	2.12 ± 0.03**	1.47 ± 0.01**
total terpenes	37.11 ± 1.79	24.00 ± 3.99**	28.25 ± 0.81**	57.01 ± 1.52***	33.91 ± 1.34
Volatile Phenols					
guaiacol	19.43 ± 1.87	17.58 ± 5.82	19.99 ± 1.43	49.79 ± 13.31*	22.44 ± 1.26
eugenol	14.41 ± 0.81	13.83 ± 1.10	15.88 ± 0.39*	44.45 ± 3.33***	25.27 ± 1.33***
vanillin	3.24 ± 1.00	3.82 ± 0.86	5.23 ± 0.25*	4.63 ± 1.61	8.84 ± 3.40
total volatile phenols	37.08 ± 3.61	35.23 ± 6.99	41.10 ± 1.58	98.86 ± 18.25**	56.55 ± 1.04***
Total Volatile Compounds	209906.50 ± 12690.67	116970.10 ± 10014.84***	107123.69 ± 2667.11***	219485.94 ± 18060.37	361847.90 ± 9699.51**

a ANOVA results of differences respect to control wine. C, control wine untreated with SEGs; 1/2AFD1, 12 g/L of SEGs added in the middle of alcoholic fermentation; 1/2AFD2, 24 g/L of SEGs added in the middle of alcoholic fermentation; AMFD1, 12 g/L of SEGs added after malolactic fermentation; AMFD2, 24 g/L of SEGs added after malolactic fermentation. The mean values (*n* = 4) are shown with their standard deviation. For each compound and each treated wine, significant differences according to Fisher’s LSD test are indicated respect to control wine in each wine column (*p < 0.05; **p < 0.01; ***p <0.001).

**Table 5:** Low Molecular Weight Phenolic Compounds (mg/L) of Wines Elaborated in Contact with SEGs Added and Bottling for 1 Year^a^

	C	1/2AFD1	1/2AFD2	AMFD1	AMFD2
Flavanols					
(+)-catechin	72.68 ± 2.89	61.47 ± 0.58*	55.06 ± 1.30*	45.10 ± 0.24**	42.94 ± 1.34**
(−)-epicatechin	446.41 ± 1.70	401.00 ± 12.94*	386.34 ± 7.41**	398.26 ± 1.45**	345.57 ± 2.60***
procyanidin B2	12.22 ± 0.80	b	b	b	b
total flavanols	531.31 ± 0.40	462.46 ± 13.52*	441.40 ± 8.72**	443.35 ± 1.21***	388.50 ± 1.25***
Phenolic Acids					
ellagic acid	59.95 ± 0.08	55.11 ± 0.41**	52.59 ± 0.52**	50.55 ± 1.82*	54.08 ± 0.44**
gallic acid	49.42 ± 0.52	46.04 ± 0.06*	36.34 ± 0.33**	8.65 ± 0.10***	9.88 ± 0.13***
protocatechuic acid	b	b	b	b	b
syringic acid	5.18 ± 0.20	5.68 ± 0.27	5.78 ± 0.87	7.71 ± 0.50*	5.91 ± 0.32
*t*-caffeic acid	3.87 ± 0.01	10.98 ± 0.86**	4.38 ± 0.20	b	5.38 ± 0.11**
*t*-caftaric acid^c^	20.47 ± 0.82	8.47 ± 0.46**	12.58 ± 0.18**	b	1.03 ± 0.03***
*t-*coumaric acid	b	b	b	b	b
*t*-coutaric acid^d^	6.83 ± 0.03	6.29 ± 1.10	3.59 ± 0.16**	b	b
4-hydroxybenzoic acid	2.66 ± 0.17	2.77 ± 0.07	3.14 ± 0.08	3.76 ± 0.10*	3.26 ± 0.25***
vanillic acid	2.64 ± 0.24	2.86 ± 0.19	2.04 ± 0.06	3.72 ± 0.07*	2.06 ± 0.20
total phenolic acids	151.02 ± 0.62	138.21 ± 0.80**	120.44 ± 1.74**	74.28 ± 1.05***	81.56 ± 0.53***
Stilbenes					
*t*-resveratrol	b	0.99 ± 0.04***	1.11 ± 0.10**	1.44 ± 0.11***	1.30 ± 0.05***
total stilbenes	b	0.99 ± 0.04***	1.11 ± 0.10**	1.44 ± 0.11***	1.30 ± 0.05***
Anthocyanins					
delphinidin 3-*O*-glucoside^e^	13.99 ± 0.47	14.45 ± 0.14	14.58 ± 0.69	13.77 ± 0.41	11.66 ± 0.03*
cyanidin 3-*O*-glucoside^e^	b	b	b	b	b
petunidin 3-*O*-glucoside^e^	24.76 ± 0.81	25.44 ± 0.51	26.73 ± 0.45	23.51 ± 0.35	19.91 ± 0.27*
peonidin 3-*O*-glucoside^e^	b	b	b	b	b
malvidin 3-*O*-glucoside	100.19 ± 0.58	112.37 ± 1.46**	123.33 ± 0.85***	105.03 ± 1.84	103.34 ± 1.33
malvidin 3-(6′-acetyl)-glucoside^e^	8.44 ± 0.05	8.73 ± 0.22	9.40 ± 0.09**	8.42 ± 0.47	8.74 ± 0.17
malvidin 3-(6′*-t*-caffeoyl)-glucoside^e^	4.94 ± 0.08	4.74 ± 0.14	5.19 ± 0.20	4.66 ± 0.12	4.57 ± 0.24
cyanidin 3-(6′-*p*-coumaroyl)-glucoside^e^	b	b	b	b	b
petunidin 3-(6′-*p*-coumaroyl)-glucoside^e^	5.89 ± 0.30	5.86 ± 0.14	6.10 ± 0.23	5.43 ± 0.06	4.94 ± 0.13
malvidin 3-(6′-*p*-coumaroiy)-glucoside^e^	18.40 ± 0.17	18.71 ± 0.11	22.38 ± 0.09**	15.52 ± 0.21**	11.15 ± 0.17***
total anthocyanins	176.62 ± 0.26	190.30 ± 1.79**	207.71 ± 1.38***	176.33 ± 1.58	164.31 ± 2.09*
Flavonols					
myricetin 3-*O*-galactoside^f^	b	b	b	b	b
myricetin 3-*O*-glucurunide + myricetin 3-*O*-glucoside^f^	2.31 ± 0.06	1.89 ± 0.00**	1.79 ± 0.06*	1.78 ± 0.13*	1.60 ± 0.04**
quercetin 3-*O*-glucuronide/glucoside^f^	b	b	b	b	b
laricitrin 3-*O*-glucuronide/galactoside^f^	b	b	b	b	b
syringetin 3-*O*-glucoside^f^	2.49 ± 0.05	2.11 ± 0.05*	2.13 ± 0.11	2.12 ± 0.00**	1.70 ± 0.03**
myricetin^f^	5.54 ± 0.04	3.29 ± 0.09**	2.99 ± 0.07***	2.52 ± 0.01***	2.14 ± 0.00***
quercetin^f^	4.84 ± 0.17	2.83 ± 0.04**	2.49 ± 0.03***	2.50 ± 0.03**	1.67 ± 0.10**
total flavonols	15.18 ± 0.13	10.11 ± 0.18***	9.40 ± 0.05***	8.92 ± 0.11***	7.12 ± 0.17***
Total Phenolic Compounds	874.13 ± 0.10	802.07 ± 11.16*	780.06 ± 5.74**	704.32 ± 1.42***	642.79 ± 0.42***

a ANOVA results of differences respect to control wine. C, control wine untreated with SEGs; 1/2AFD1, 12 g/L of SEGs added in the middle of alcoholic fermentation; 1/2AFD2, 24 g/L of SEGs added in the middle of alcoholic fermentation; AMFD1, 12 g/L of SEGs added after malolactic fermentation; AMFD2, 24 g/L of SEGs added after malolactic fermentation. The mean values (*n* = 4) are shown with their standard deviation. For each compound and each treated wine, significant differences according to Fisher’s LSD test are indicated respect to control wine in each wine column (*p < 0.05; **p < 0.01; ***p <0.001).

b Not detected.

c *t*-Caftaric acid was quantified in terms of *t*-caffeic acid.

d *t*-Coutaric acid was quantified in terms of *t*-coutaric acid.

e Anthocyanins were quantified in terms of malvidin 3-*O*-glucoside.

f Flavonols were quantified in terms of quercetin 3-*O*-glucoside.

### Sensorial Wine Evolution

To ascertain the effect of the two main factors considered to obtain the different wines in this study (dose and moment of addition of SEGs during winemaking) on the sensorial descriptors throughout bottle aging, a multivariate analysis (MANOVA) was performed, whereby the different scores of each wine were compared to the control. For this, the sensory sampling tastings conducted at bottling, after 4 months, and after 1 year were considered; the results are summarized in Table 1. Among the individual factors considered, “moment” of addition had the most significant effect on almost all of the analyzed descriptors (Table 1), except for garnet, red fruits (olfactory phase), red fruits, green character (taste phase), and bitterness tannin. However, the “dose” factor was found to influence only certain descriptors, such as red fruits and toasted at the olfactory and taste phases, respectively, and silkiness tannin. Regarding the two-way interaction (“dosage × moment”), the effect was significant for the red fruits and SEG descriptors, both at olfactory and taste phase. These results show that the moment at which SEGs were added during the winemaking was decisive for the sensorial perception of differences with respect to the control wine.

**Figure 1:**
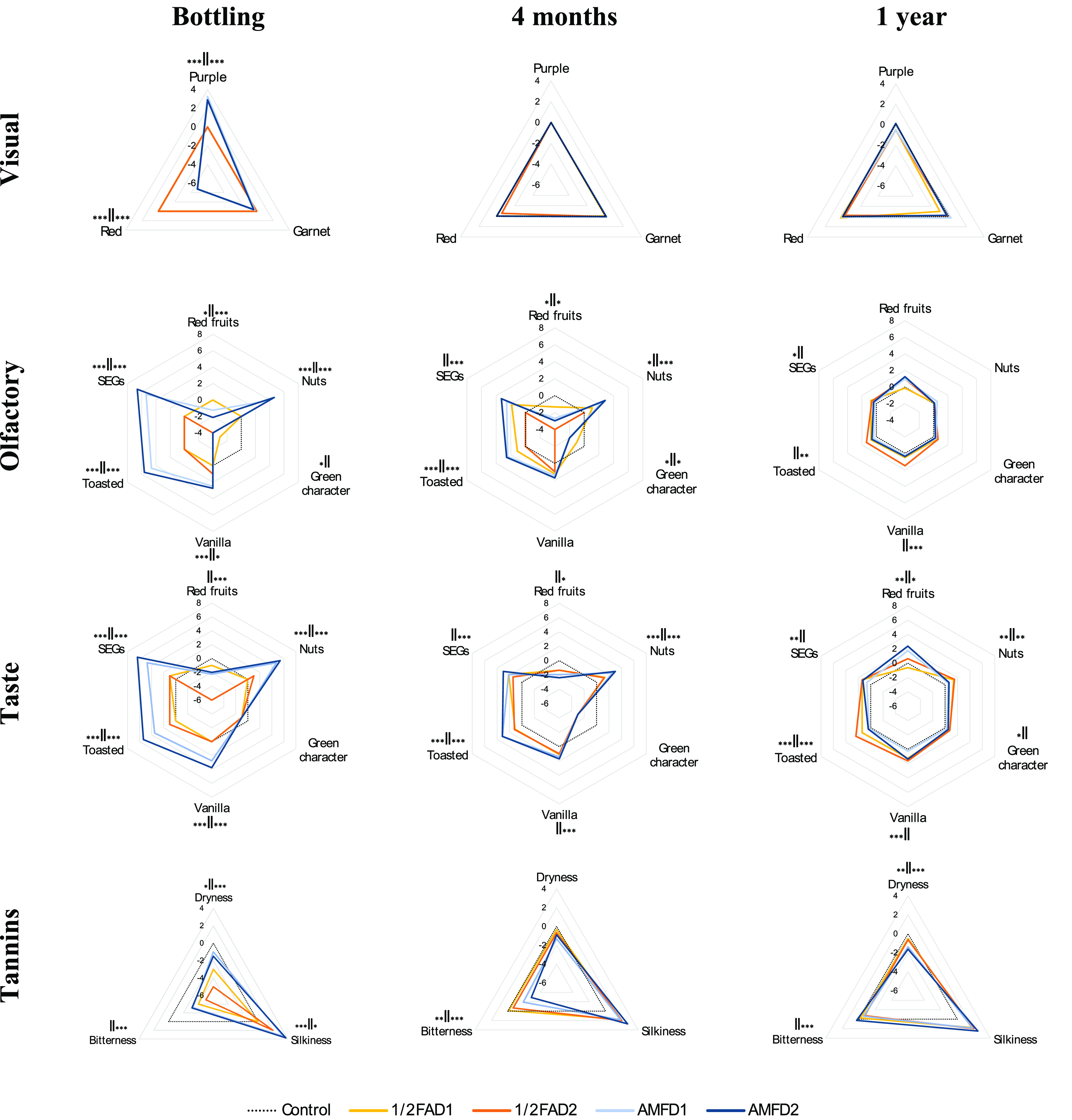
Sensorial analysis descriptor mean scores for the differences with respect to the control wine at bottling, 4 months, and 1 year. 1/2AFD1, 12 g/L of SEGs added in middle of alcoholic fermentation; 1/2AFD2, 24 g/L of SEGs added in middle of alcoholic fermentation; AMFD1, 12 g/L of SEGs were added after malolactic fermentation; AMFD2, 24 g/L of SEGs were added after malolactic fermentation. For each descriptor, significant differences between the differences with respect to the control of the wines obtained with the same dose (D1 or D2) but at a different time (1/2AF and AMF) are indicated according to Fisher’s LSD test (*p < 0.05; **p < 0.01; ***p < 0.001) and attending to the schema: significant difference for D1 dose∥significant difference for D2 dose.

The sensorial descriptors studied for the three sampling moments indicated (bottling, 4 months, and 1 year) are presented in Figure 1. The descriptors have been grouped as visual, olfactory, taste, and tannin phases. As the “Moment” factor was revealed to have the most significant effect on the perception of sensorial descriptors, the one-way analysis of variance (ANOVA) showed the significant differences obtained using the same dose but added at a different time (1/2AF, SEGs added in the middle of alcoholic fermentation; AMF, SEGs added after malolactic fermentation). Positive values indicate a higher perception of the attribute with respect to control, while negative values indicate a lower perception of the attribute with respect to control. The zero line (dashed line) on the spider charts (Figure 1) shows the location of the normalized control wine.

The greatest differences were found at bottling time, while, as the time in the bottle progressed, the differences in scores given for each attribute decreased compared to the control wine. This decrease in the difference in scores was less pronounced during the second period of storage. This first notable impact on the wines at the end of the period of contact with SEGs is consistent with what happens when other types of oak products alternative to the barrel are used, such as chips.^21^ In the wines from bottling sampling moment, the aromas released by the SEGs were not well integrated with the wines’ other aromatic compounds; therefore, it could be concluded that the aromas released by the SEGs are perceived as more aggressive with rough edges that mask other nuances typical of the wines of the variety, such as the red fruits, as shown in Figure 1 (lower values for olfactory and taste phases compared to control). These differences were greater for the D2 dose.

Significant differences were found in the visual valuation of the wines at bottling time, related to the purple and red descriptors. Wines from both doses of the AMF moment of addition presented greater differences with respect to the control than the wines from 1/2AF with regard to the purple descriptor. These wines showed red values lower than the control, which were significantly different for both doses (Figure 1). These differences in AMF wines could be due to a copigmentation reaction, given that this phenomenon usually produces an increase in absorbance (hyperchromic effect) and a positive shift of the visible absorption maximum (bathochromic effect). In addition to the contribution of free anthocyanins, the copigmentation phenomenon influences the color of young red wines;^22^ this is due to the first interaction between the anthocyanins and other wine components, which leads to the formation of new colored compounds during red wine aging.^23^ Copigmentation depends on the structure of the anthocyanins and particularly on the structure of the copigments. Flavonols have greater potential than flavan-3-ols to act as copigments, but red wine is more concentrated in flavan-3-ols than in flavonols and, within that, (−)-epicatechin is a better copigment due to its C ring conformation, allowing it to be approximately coplanar.^24^ In fact, the (−)-epicatechin content in these wines was significantly lower than the corresponding control wine.^10^ Such differences were not observed in subsequent tastings (4 months and 1 year). This may be because copigmentation decreases during storage while the concentration of polymerized structures increases, with consequent changes in wine color from red purple to brick red hues, which has been attributed to the progressive formation of new pigments, given that anthocyanins react with other compounds.^25,26^ Despite this decrease, the increase in brick red hues was not significant; hence this was not considered a descriptor.

For the olfactory phase, the SEGs’ imprint was evident from the positive values for the nuts, toasted, and vanilla descriptors (Figure 1) at the bottling tasting time. In addition, a term associated with this enological additive, a descriptor called SEGs, has been considered. This descriptor would indicate sweet wood, as a result of the proposal by the authors of a similar odorant series, who suggested “sweet woody” for the SEG character.^10^ For the four descriptors (nuts, toasted, vanilla, and SEGs), statistically significant differences were observed between the two addition moments in the two doses. A decrease was observed in the red fruits descriptor, which was more pronounced in the highest dose of SEGs. This factor could be due to the contribution of different phenomena: on one hand, the enrichment of wine with volatile compounds released by SEGs may have a masking effect on fruity odor when present at suprathreshold concentrations^27^ but a synergistic effect if present at subthreshold and perithreshold levels. On the other hand, the adsorption of fruity esters, alcohols, and acetates by wood may occur to varying extents dependent on the wine and wood composition, as previous works have reported.^28,29^ The volatile composition of SEG wines revealed a slight decrease in some compounds, such as β-damascenone, farnesol, diethyl succinate, and ethyl acetate.^10^ Further, after 4 months of bottle aging, red fruits were less perceptible in the control wines (positive values for treated wines), which coincides with the significant reduction of the volatile compounds diethyl succinate, ethyl acetate, isoamyl acetate, hexyl acetate, and farnesol related to this odorant series (Table 2). In the last sensory tasting (1 year), the red fruits were perceived slightly more in the AMF wines (SEGs added after malolactic fermentation), but not enough to show significant differences (Table 4). From the volatile point of view, the lower significant concentration of some compounds remained (ethyl butyrate, diethyl succinate, and ethyl acetate) (Table 4), while other compounds related to this descriptor showed significantly higher content in AMF wines (e.g., farnesol, benzyl alcohol, ethyl octanoate, ß-damascenone, and hexyl acetate) (Table 4), which would lead to a greater impact of the descriptor red fruits. Nevertheless, knowledge of volatile composition and concentration alone is not enough to completely understand the flavor of wines, as some interactions among odorants, sense modalities, and matrix effects can impact the odorant volatility, aroma release, and overall perceived aroma intensity and quality. Additionally, the treated wines showed a less green character than the control at bottling time (negative values), which was significantly different between the addition moments when the lower dose was used. However, the compounds related to green character (1-hexanol and 3-hexen-1-ol) were of higher concentration in the treated wines^10^ at both 4 months and 1 year (Tables 2 and 4), therefore, this difference compared to the control was due to other compounds and was not determined by the method used. After 4 months, these last differences extended to the highest dose as well, suggesting that the SEGs could have a direct influence regarding the reduction of the green character of the wines, which constitutes one of the problems that most worries the sector concerning detraction from quality. According to Sáenz-Navajas et al.,^30^ this character is linked to the vegetable aroma, astringency, and drying tannins. The descriptors most related to contact with wood (nuts, vanilla, toasted, and SEGs) continued to be higher than the control (positive values), but with differences that were not as pronounced as at bottling time. However, these wood-related descriptors maintained significantly higher values for total volatile phenols in the AMF wines, mainly due to the increase in eugenol (Table 2). Adding to the fact that the differences found between the two moments of addition for the two doses were less significant, this behavior revealed an improved integration of the SEGs’ character, leading to the reduction of the rough edges mentioned in the initial tasting. After 1 year, the SEGs’ character was notable between the two addition moments only at the lowest dose, while for the highest, only the vanilla and toasted descriptors showed significant differences.

In relation to the taste phase, the descriptors studied were the same as those examined for the olfactory phase (Figure 1), with the spider charts indicating that the profiles in the taste and olfactory phases are very similar. Once again, the impact from the SEGs (nuts, vanilla, toasted, and SEGs descriptors) was more notable in the sensory tasting at bottling time, whereby the two addition moments showed statistically significant differences for the two doses (Figure 1). However, the perception of red fruit was significantly different only for the highest dose. Such differences were maintained in the sensory analysis at 4 months for the highest dose. However, after 1 year of bottle aging, the differences between the moments of addition of the SEGs were more noticeable at the highest dose, including for red fruit and green character (Figure 1).

Related to the tannin perception, at the tasting time for the bottling moment, the AMF wines were perceived as silkier compared to the 1/2AF wines, where significant differences were observed among both doses, with these variances greater at a lower dose of SEGs. In addition, the dryness and bitterness descriptors received lower scores (negative values) (Figure 1) than the control wines, with the highest dose of SEGs presenting significant differences for both descriptors between the two addition moments. The tannin composition of SEGs is characterized by condensed tannin (proanthocyanidins),^6^ which has been shown to impact bitterness in wine, as have flavanol monomers.^31^ In this study, contact with SEGs did not lead to an increase in these compounds;^10^ therefore, the significant decrease of flavanols would explain this lower perception of bitterness by the panelists in the wines treated with SEGs. The lower concentrations of flavanols can be justified in part by the copigmentation phenomenon and the sorption of these compounds by the vine shoots.^32^ According to Ferrero-del-Teso et al.,^33^ alongside tannins’ role in the perception of dryness, certain anthocyanins have demonstrated a relevant implication in the “dry” attribute. Wines from the SEG treatment in this study showed lower concentrations in some of the anthocyanins analyzed, which corroborates Ferrero-del-Teso’s research findings.

After 4 months of bottle aging, the differences with respect to the control wine in the bitterness descriptor were maintained and also the lower significant values of flavanol compounds (Table 3). Further, wines from the treatments were silkier again, although there were no statistically significant differences in the doses used between the two moments. The same was observed for the dryness descriptor; therefore, SEGs could be considered accelerators to eliminate initial bitterness and dryness from wines. After 1 year, the wines treated with SEGs maintained lower dryness than the control, with differences shown between the two moments of addition for both doses, and less bitterness than the control, with statistically significant differences for the higher dose (Figure 1).

### Adjustment of the SEG Sensorial Descriptor Evolution

The results from the sensorial analysis for the three sampling moments considered (bottling, 4 months, and 1 year; Figure 1) showed that the evolution of the wines was greater in the first 4 months after bottling. During this period, the sensorial analysis was conducted monthly with the aim of highlighting the trend of the descriptors most related to the impact of the SEG use. Figure S1 (supplementary data) shows the mean values of the differences compared to the control for each of the sampling moments, from bottling to 4 months. Adjusting the evolution of the sensorial descriptors is complex; however, given the profile the results showed and after attempting various adjustments to enable equal comparison, the most effective adjustment found was linear regression. Table 6 shows the values of the adjustment parameters of the linear regression (slope, intercept, and *R*^2^) associated with the four treatments with vine shoots for the descriptors nuts, vanilla, toasted, and SEGs, in both the olfactory and taste phase, as well as silkiness. Considering that the addition moment of SEGs has been shown to be the most influential factor on the sensorial characteristics of the wines (Table 1), comparisons were made between the moments of addition (1/2AF and AMF) for each of the applied doses (D1 and D2). The data obtained for the statistical differences for slope and intercept values are shown in Table 6. The *R*^2^ values (proportion of the total variance of the variable explained by the regression) were quite different, which once again depicts the complexity of fit for these parameters. However, these values were found to be acceptable (0.75 to 0.90), good (0.90 to 0.95), and very good (0.95 to 1), depending on the descriptor and the treatment. The best fit was observed for the nuts and toasted descriptors (olfactory phase) in the 1/2AFD2 wines and for SEGs (taste phase) in both doses of the AMF wines (Table 6).

**Table 6:** Values of the Fitting Parameters of the Linear Regression Associated with the Four Vine Shoot Treatments during Winemaking for the Descriptors Related to the SEG Impact^a^

				D1		D2	
				1/2AF	AMF	1/2AF	AMF
olfactory	nuts	slope	value	–0.0151	–0.0124	–0.0160	–00151
			*p*-value				
		intercept	value	2.75	3.672	2.945	4.406
			*p*-value	*		**	
			*R*^2^	0.799	0.627	0.978	0.847
	vanillin	slope	value	0.0009	–0.007	–0.0053	–0.0069
			*p*-value				
		intercept	value	1.235	2.258	1.75	2.32
			*p*-value	*			
			*R*^2^	0.076	0.691	0.443	0.453
	toasted	slope	value	–0.0106	–0.019	–0.0145	–0.0245
			*p*-value				
		intercept	value	2.445	4.442	2.89	5.302
			*p*-value	**		**	
			*R*^2^	0.899	0.852	0.985	0.760
	SEGs	slope	value	–0.0047	–0.0221	–0.0025	–0.0289
			*p*-value	*		*	
		intercept	value	2.465	5.034	2.585	6.22
			*p*-value	**		**	
			*R*^2^	0.319	0.928	0.051	0.887
taste	nuts	slope	value	–0.0135	–0.0167	–0.0295	–0.0185
			*p*-value				
		intercept	value	2.91	4.162	4.52	4.854
			*p*-value	*		*	
			*R*^2^	0.927	0.642	0.819	0.777
	vanillin	slope	value	–0.0101	–0.0081	–0.0211	–0.0125
			*p*-value				
		intercept	value	2.13	2.37	3.165	2.89
			*p*-value				
			*R*^2^	0.600	0.415	0.729	0.361
	toasted	slope	value	–0.0150	–0.0102	–0.0122	–0.0227
			*p*-value				
		intercept	value	3.355	3.754	2.855	5.202
			*p*-value			*	
			*R*^2^	0.599	0.309	0.590	0.736
	SEGs	slope	value	–0.0086	–0.0233	–0.0168	–0.0300
			*p*-value	*			
		intercept	value	3.03	5.066	3.715	6.378
			*p*-value	**		***	
			*R*^2^	0.766	0.950	0.773	0.978
	silkiness	slope	value	–0.0171	–0.0146	–0.0054	–0.0091
			*p*-value				
		intercept	value	3.6	3.664	2.478	3.74
			*p*-value			*	
			*R*^2^	0.818	0.772	0.165	0.806

a D1, 12 g/L of SEGs; D2, 24 g/L of SEGs; 1/2AF, SEGs added in the middle of alcoholic fermentation; AMF, SEGs added after malolactic fermentation. Significant differences according to Fisher’s LSD test are indicated for each dose (D1 or D2) between the two different times of addition (1/2AF and AMF) (*p < 0.05; **p < 0.01; ***p <0.001).

Related to the slope values, the tendency for all descriptors was a drop in their perception (negative values), which was, in general, greater for wines in which the SEGs were added after malolactic fermentation (AMF). However, this trend was only statistically significant (*p* <0.05) for the SEGs descriptor in D1, in both the olfactory and taste phases, and for the SEGs olfactory phase in D2 (Table 6). Interestingly, for the silkiness descriptor, the highest slope values were observed for the lowest dose in both wines (1/2AF and AMF), which implies that a higher dose means that silkiness in wines is appreciated for a longer time. Regarding the intercepts, these were higher for wines from D2, regardless of the time of addition of SEGs (1/2AF and AMF), which shows that the impact is greater the higher the dose used. In addition, the lack of significant differences for most of the descriptors in the slope values indicates that the reduction in the perception of these descriptors responds to the same pattern, regardless of the dose used.

### Effect of SEG Treatments during Winemaking on Differentiation throughout Bottle Aging

**Figure 2:**
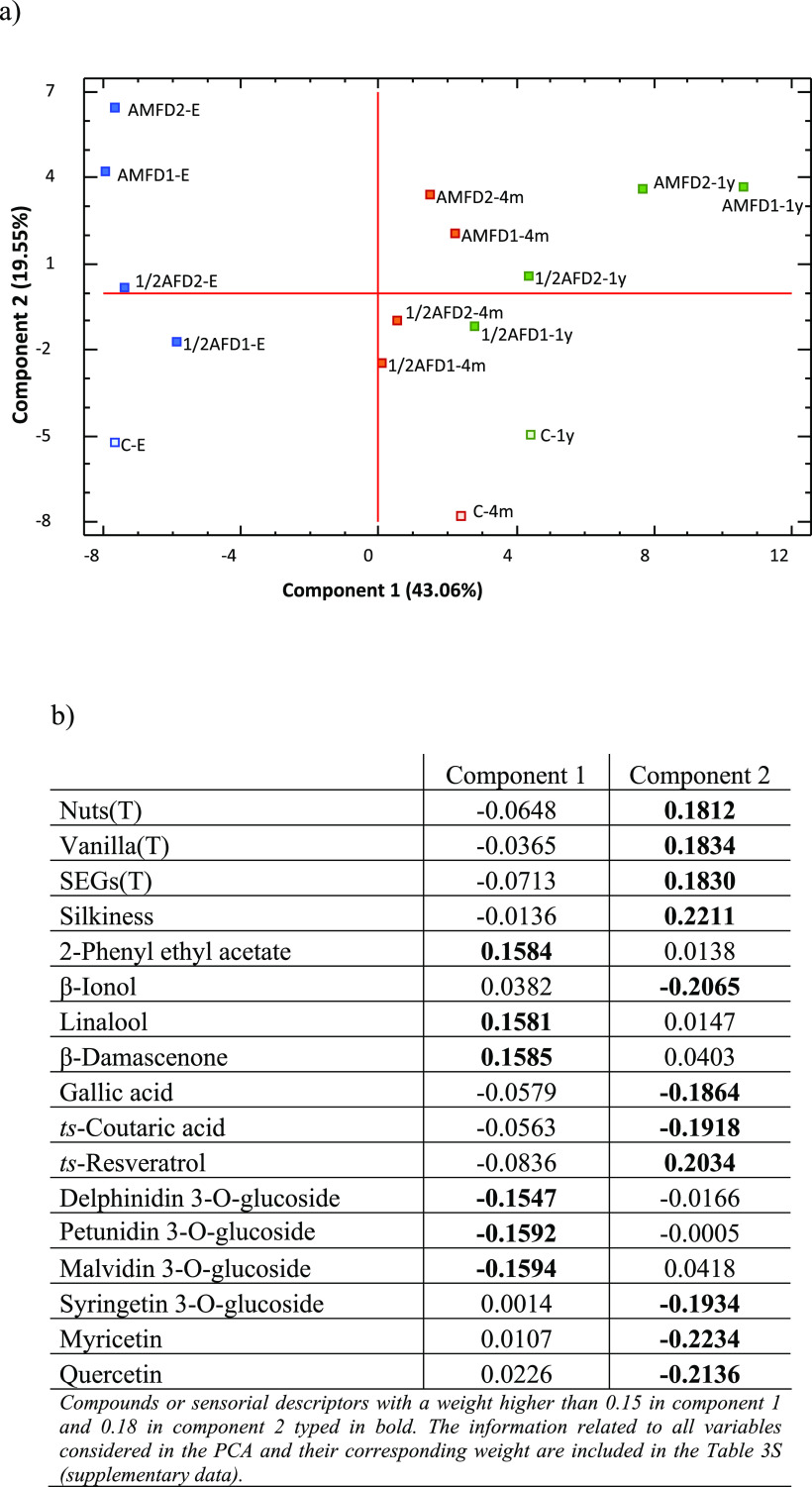
Principal component analysis (PCA) of wines at the time of bottling, 4 months, and 1 year performed with volatile and phenolic composition and sensorial analysis results. (a) Projection of wine samples in the plane formed by the two main components. (b) Weights of the variables most influential in the first two principal components.

To obtain a reduced number of linear combinations of the variables that explain the greater variability in the data, a principal component analysis (PCA) was conducted, using the data outlined in Tables 2–5 in this study as well as from the research of Cebrián-Tarancón et al.^10^ related to volatile and phenolic composition and sensorial analysis (Figure 1), which all of them were previously normalized. The results are shown in Figure 2. The first three components obtained explain 76.55% of the variability in the original data, where the first main component (PC1) encompassed 43.06% of that total, the second (PC2) 19.55%, and the third (PC3) 13.94% (Figure 2a), with 2-phenylethyl acetate, linalool, β-damascenone, delphinidin 3-*O*-glucoside, petunidin 3-*O*-glucoside, and malvidin 3-*O*-glucoside being the variables with the greatest weight in component 1 and nuts, vanilla, SEGs (from taste phase), silkiness, β-ionol, gallic acid, *trans*-coutaric acid, *trans*-resveratrol, syringetin 3-*O*-glucoside, myricetin, and quercetin comprising component 2 (Figure 2b). Projection of the wines on the factor-plane showed that wines were positioned according to their bottle aging time (Figure 2a) and demonstrated the significance of the variables in the wines regarding bottle aging time (Figure 2b). There was more distance between the first and second samplings, which indicates a greater evolution between bottling and 4 months compared to other time periods, while there was almost no differentiation between 4 months and 1 year of bottled aging. The first main component, PC1, enables differentiation between the wines at the time of bottling, after 4 months, and after 1 year and contains information on volatile composition, mainly terpenes and norisoprenoids and the major monomeric anthocyanins, which contribute to the separation to a greater extent. The second main component, PC2, enables differentiation between control wines and treatments and, within the latter, differentiation between the moments of addition of SEGs (1/2AF and AMF wines); PC2 is primarily defined by descriptors of the taste phase related to the impact of the SEGs, together with phenolic compounds (flavanols, phenolic acids, and stilbenes). According to the distribution of the wines, those at bottling time were located in the negative PC1 and logically defined by the monomeric anthocyanins, showing significant levels of compounds responsible for their red color despite the fact that the use of SEGs implies a reduction in the color of the wines.^32^ After 4 months of bottle aging, the wines had significant levels of terpenes and norisoprenoids, which are responsible for the transfer of the SEGs. In the following 8 months, the importance of the variables was maintained. On the other hand, considering PC2, wines in which the SEGs were added at the end of the malolactic fermentation (AMF) were located on the positive axis, showing a greater impact of the taste phase descriptors related to the use of the SEGs, regardless of the time of sampling. The control wines can be found toward the PC2 negative part and are characterized by higher contents of phenolic compounds and flavanols, which was to be expected given this content was higher in those wines (Tables 2 and 4).

In conclusion, the use of toasted vine shoots as an alternative enological tool is an innovative practice to differentiate wines that has led to the establishment of a new associated descriptor, SEGs, which is related to “sweet wood” character.

The results of this examination of the bottle aging of Tempranillo wines with different doses of SEGs (12 and 24 g/L) and addition at varied winemaking moments showed the moment of addition to be the factor most affecting the sensorial descriptors. This first impact due to the SEGs was that the related descriptors (nuts, vanilla, toasted, and SEGs) were perceived as more aggressive with rough edges, masking other nuances typical of Tempranillo wines. However, as bottle aging progressed, these differences gradually smoothed out, resulting in more rounded wines, where the nuances due to SEGs were better integrated.

The greatest differences between the wines, concerning chemical composition (volatile and phenolic compounds) and sensory analysis, were found between bottling and 4 months of bottle aging. During this period, the evolution of the descriptors related to the use of SEGs showed a linear downward trend, where the SEGs descriptor had the best values in the adjustment.

In view of these results, it can be concluded that, for wines treated with SEGs, bottle aging after treatment during winemaking is crucial so that the notes from the SEGs provide balanced complexity in the wines, thereby enriching and differentiating them.

## Supporting Information Available

Linear range, LOD, LOQ, %RSD, and recovery for volatile compounds and phenolic compounds, weights of the total variables (volatile and phenolic compounds, and sensorial descriptors) in the first two principal components, and linear regression for the differences with respect to the control wine, associated with the four vine shoot treatments during winemaking for the descriptors related to the SEG impact (PDF)

## References

[ref1] Costa J. M.; Catarino S.; Escalona J. M.; Comuzzo P. Achieving a More Sustainable Wine Supply Chain—Environmental and Socioeconomic Issues of the Industry. Improv. Sustain. Vitic. Winemak. Pract. 2022, 1–24. 10.1016/B978-0-323-85150-3.00009-8.

[ref2] Teissedre P. L.; Catarino S.; Comuzzo P. Wine Quality Production and Sustainability. Improv. Sustain. Vitic. Winemak. Pract. 2022, 187–199. 10.1016/B978-0-323-85150-3.00005-0.

[ref3] Calderón-Martín M.; Valdés-Sánchez E.; Alexandre-Franco M. F.; Fernández-González M. C.; Vilanova de la Torre M.; Cuerda-Correa E. M.; Gómez-Serrano V. Waste Valorization in Winemaking Industry: Vine Shoots as Precursors to Optimize Sensory Features in White Wine. LWT 2022, 163, 113601 10.1016/j.lwt.2022.113601.

[ref4] Martínez-Gil A. M.; del Alamo-Sanza M.; Nevares I.; Sánchez-Gómez R.; Gallego L. Effect of Size, Seasoning and Toasting Level of Quercus Pyrenaica Willd. Wood on Wine Phenolic Composition during Maturation Process with Micro-Oxygenation. Food Res. Int. 2020, 128, 108703 10.1016/j.foodres.2019.108703. 31955781

[ref5] Cebrián-Tarancón C.; Fernández-Roldán F.; Sánchez-Gómez R.; Salinas R.; Llorens S. Vine-Shoots as Enological Additives. A Study of Acute Toxicity and Cytotoxicity. Foods 2021, 10 (6), 1267 10.3390/foods10061267. 34199530 PMC8226571

[ref6] Cebrián-Tarancón C.; Sánchez-Gómez R.; Gómez-Alonso S.; Hermosín-Gutierrez I.; Mena-Morales A.; García-Romero E.; Salinas M. R.; Zalacain A. Vine-Shoot Tannins: Effect of Post-Pruning Storage and Toasting Treatment. J. Agric. Food Chem. 2018, 66 (22), 5556–5562. 10.1021/acs.jafc.8b01540. 29770693

[ref7] Cebrián-Tarancón C.; Sánchez-Gómez R.; Oliva J.; Cámara M. A.; Zalacain A.; Salinas M. R. M. R. Evolution of Fungicide Residues in Pruned Vine-Shoots. Oeno One 2021, 55 (1), 145–152. 10.20870/oeno-one.2021.55.1.4488.

[ref8] Cebrián-Tarancón C.; Fernández-Roldán F.; Alonso G. L.; Salinas R. M. Classification of Vine-Shoots for Use as Enological Additives. J. Sci. Food Agric. 2022, 102, 724 10.1002/jsfa.11403. 34171125

[ref9] Fanzone M.; Catania A.; Assof M.; Jofré V.; Prieto J.; Quiroga D. G.; Sottano J. L.; Sari S. Application of Vine-shoot Chips during Winemaking and Aging of Malbec and Bonarda Wines. Beverages 2021, 7 (3), 51 10.3390/beverages7030051.

[ref10] Cebrián-Tarancón C.; Fernández-Roldán F.; Sánchez-Gómez R.; Alonso G. L.; Salinas M. R. Pruned Vine-Shoots as a New Enological Additive to Differentiate the Chemical Profile of Wines. Food Res. Int. 2022, 156, 111195 10.1016/j.foodres.2022.111195. 35651049

[ref11] Cebrián-Tarancón C.; Sánchez-Gómez R.; Cabrita M. J.; García R.; Zalacain A.; Alonso G. L.; Salinas M. R. Winemaking with Vine-Shoots. Modulating the Composition of Wines by Using Their Own Resources. Food Res. Int. 2019, 121, 117–126. 10.1016/j.foodres.2019.03.032. 31108731

[ref12] Cebrián-Tarancón C.; Sánchez-Gómez R.; Carot J. M.; Zalacain A.; Alonso G. L.; Salinas M. R. Assessment of Vine-Shoots in a Model Wines as Enological Additives. Food Chem. 2019, 288, 86–95. 10.1016/j.foodchem.2019.02.075. 30902319

[ref13] Cebrián-Tarancón C.; Sánchez-Gómez R.; Salinas M. R.; Alonso G. L.; Oliva J.; Zalacain A. Toasted Vine-Shoot Chips as Enological Additive. Food Chem. 2018, 263 (April), 96–103. 10.1016/j.foodchem.2018.04.105. 29784334

[ref14] Guadalupe Z.; Ayestarán B. Changes in the Color Components and Phenolic Content of Red Wines from Vitis Vinifera L. Cv. “Tempranillo” during Vinification and Aging. Eur. Food Res. Technol. 2008, 228 (1), 29–38. 10.1007/s00217-008-0902-2.

[ref15] Sáenz-Navajas M. P.; Avizcuri J. M.; Ferreira V.; Fernández-Zurbano P. Sensory Changes during Bottle Storage of Spanish Red Wines under Different Initial Oxygen Doses. Food Res. Int. 2014, 66, 235–246. 10.1016/j.foodres.2014.08.053.

[ref16] He J.; Zhou Q.; Peck J.; Soles R.; Qian M. C. The Effect of Wine Closures on Volatile Sulfur and Other Compounds during Post-Bottle Ageing. Flavour Fragr. J. 2013, 28 (2), 118–128. 10.1002/ffj.3137.

[ref17] Gómez Gallego M. A.; Gómez García-Carpintero E.; Sánchez-Palomo E.; González Viñas M. A.; Hermosín-Gutiérrez I. Evolution of the Phenolic Content, Chromatic Characteristics and Sensory Properties during Bottle Storage of Red Single-Cultivar Wines from Castilla La Mancha Region. Food Res. Int. 2013, 51 (2), 554–563. 10.1016/j.foodres.2013.01.010.

[ref18] O’Brien V.; And L. F.; Ozidac P. Packaging Choices Affect Consumer Enjoyment of Wine. Wine Vitic. J. 2009, (5), 48–54.

[ref19] International Organisation of Vine and Wine. Compendium of International Methods of Wine and Must Analysis; OIV: Paris, France, 2014.

[ref20] Sánchez-Gómez R.; Zalacain A.; Alonso G. L.; Salinas M. R. Effect of Toasting on Non-Volatile and Volatile Vine-Shoots Low Molecular Weight Phenolic Compounds. Food Chem. 2016, 204, 499–505. 10.1016/j.foodchem.2016.02.137. 26988529

[ref21] Santos F.; Correia A. C.; Ortega-Heras M.; García-Lomillo J.; González-SanJosé M. L.; Jordão A. M.; Ricardo-da-Silva J. M. Acacia, Cherry and Oak Wood Chips Used for a Short Aging Period of Rosé Wines: Effects on General Phenolic Parameters, Volatile Composition and Sensory Profile. J. Sci. Food Agric. 2019, 99 (7), 3588–3603. 10.1002/jsfa.9580. 30628096

[ref22] He F.; Liang N. N.; Mu L.; Pan Q. H.; Wang J.; Reeves M. J.; Duan C. Q. Anthocyanins and Their Variation in Red Wines II. Anthocyanin Derived Pigments and Their Color Evolution. Molecules 2012, 17 (2), 1483–1519. 10.3390/molecules17021483. 23442981 PMC6269080

[ref23] Fernandes A.; Oliveira J.; Teixeira N.; Mateus N.; De Freitas V. A Review of the Current Knowledge of Red Wine Colour. Oeno One 2017, 51 (1), 1604 10.20870/oeno-one.2017.51.1.1604.

[ref24] Brouillard R.; Wigand M. C.; Dangles O.; Cheminat A. PH and Solvent Effects on the Copigmentation Reaction of Malvin with Polyphenols, Purine and Pyrimidine Derivatives. J. Chem. Soc., Perkin Trans. 1991, 2 (8), 1235–1241. 10.1039/p29910001235.

[ref25] Es-Safi N. E.; Cheynier V. Flavanols and Anthocyanins as Potent Compounds in the Formation of New Pigments during Storage and Aging of Red Wine. ACS Symp. Ser. 2004, 886, 143–159. 10.1021/bk-2004-0886.ch009.

[ref26] Fulcrand H.; Dueñas M.; Salas E.; Cheynier V. Phenolic Reactions during Winemaking and Aging. Am. J. Enol. Vitic. 2006, 57 (3), 289 10.5344/ajev.2006.57.3.289.

[ref27] Atanasova B.; Thomas-Danguin T.; Langlois D.; Nicklaus S.; Etievant P. Perceptual Interactions between Fruity and Woody Notes of Wine. Flavour Fragr. J. 2004, 19 (6), 476–482. 10.1002/ffj.1474.

[ref28] Coelho E.; Domingues L.; Teixeira J. A.; Oliveira J. M.; Tavares T. Understanding Wine Sorption by Oak Wood: Modeling of Wine Uptake and Characterization of Volatile Compounds Retention. Food Res. Int. 2019, 116, 249–257. 10.1016/j.foodres.2018.08.025. 30716943

[ref29] Ramirez Ramirez G.; Lubbers S.; Charpentier C.; Feuillat M.; Voilley A.; Chassagne D. Aroma Compound Sorption by Oak Wood in a Model Wine. J. Agric. Food Chem. 2001, 49 (8), 3893–3897. 10.1021/jf001334a. 11513685

[ref30] Sáenz-Navajas M. P.; Arias I.; Ferrero-del-Teso S.; Fernández-Zurbano P.; Escudero A.; Ferreira V. Chemo-Sensory Approach for the Identification of Chemical Compounds Driving Green Character in Red Wines. Food Res. Int. 2018, 109, 138–148. 10.1016/j.foodres.2018.04.037. 29803435

[ref31] Sáenz-Navajas M. P.; Avizcuri J. M.; Ballester J.; Fernández-Zurbano P.; Ferreira V.; Peyron D.; Valentin D. Sensory-Active Compounds Influencing Wine Experts’ and Consumers’ Perception of Red Wine Intrinsic Quality. LWT - Food Sci. Technol. 2015, 60 (1), 400–411. 10.1016/j.lwt.2014.09.026.

[ref32] Cebrián-Tarancón C.; Fernández-Roldán F.; Sánchez-Gómez R.; Alonso G. L.; Salinas M. R. Chemical Exchange in the Vine Shoots-Wine System When Used as an Innovative Enological Procedure. J. Sci. Food Agric. 2023, 103, 1821 10.1002/jsfa.12338. 36377405 PMC10107323

[ref33] Ferrero-del-Teso S.; Suárez A.; Jeffery D. W.; Ferreira V.; Fernández-Zurbano P.; Sáenz-Navajas M. P. Sensory Variability Associated with Anthocyanic and Tannic Fractions Isolated from Red Wines. Food Res. Int. 2020, 136, 109340 10.1016/j.foodres.2020.109340. 32846535

