# Supplementary material for: Toasted Vine Shoots as an Alternative Enological Tool. Impact on the Sensory Profile of Tempranillo Wines during Bottle Aging
Source: J Agric Food Chem. 2023 Mar 24;72(4):1914–27. doi: 10.1021/acs.jafc.2c08982 (PMC10835734; doi:10.1021/acs.jafc.2c08982)
Supplement: Supplementary file 1 — jf2c08982_si_001.pdf [file jf2c08982_si_001.pdf]

SUPPORTING INFORMATION

Table S1. Linear range, LOD, LOQ, %RSD and recovery for volatile compounds.

	CAS Nº	Linear range[Δ] (µg/L)	LOD (µg/L)	LOQ (µg/L)	%RSD	%Recovery[†]
Acids						
Hexanoic acid	142-62-1	49.95-1500.63	28.09	78.53	2.55	106±8
Octanoic acid	204-677-5	60.15-1500.21	57.60	68.01	3.75	85±14
Decanoic acid	334-48-5	12.93-387.10	1.29	2.22	3.98	113±6
Alcohols						
2-Phenylethanol	60-12-8	10.21-171.43	8.07	34.99	2.81	117±8
1-Hexanol	111-27-3	5.36-999.62	3.67	12.24	0.93	101±4
cis-3-Hexen-1-ol	928-96-1	151.72-4570.39	145.19	150.19	2.01	94±3
Benzyl alcohol	100-51-6	45.45-1714.08	26.71	50.00	2.66	107±9
Nonanol	143-08-8	5.42-206.32	0.18	0.60	1.68	111±9
Aldehydes						
Benzaldehyde	100-52-7	6.27-33.76	0.77	1.00	1.39	96±7
Nonanal	124-19-6	4.96-26.72	0.71	0.97	9.55	86±9
Esters						
Ethyl lactate	97-64-3	223.72-1856.66	212.07	761.21	3.59	92±11
Ethyl octanoate	106-32-1	10.27-145.44	0.45	1.49	4.28	75±8
Ethyl butyrate	105-54-5	3.58-51.43	1.03	3.42	2.93	105±6
Ethyl decanoate	203-761-9	9.34-145.78	0.86	2.37	3.81	80±10
Diethyl succinate	123-25-1	10.71-145.27	7.13	15.20	6.19	87±5
Ethyl vanillate	617-05-0	5.25-35.84	4.99	15.85	3.77	89±6
Ethyl hexanoate	123-66-0	2.57-49.82	0.39	0.55	1.77	84±10
Ethyl cinnamate	4192-77-2	6.34-91.45	0.01	0.06	2.91	94±9
Ethyl acetate	141-78-6	36.51-541.73	14.55	19.42	7.43	101±4
Isoamyl acetate	123-92-2	1.52-22.90	0.56	1.88	8.10	97±5
2-Phenyl ethyl acetate	103-45-7	4.13-59.76	0.11	0.35	2.23	105±7
Hexyl acetate	142-92-7	3.52-51.36	0.35	0.60	1.86	107±6
Norisoprenoids						
β-Ionol	472-80-0	20.87-83.51	18.99	19.01	4.03	76±11
β-Damascenone	23726-93-4	32.05-480.92	5.50	5.69	2.56	109±1
α-Ionone	127-41-3	0.47-3.17	0.10	0.19	3.31	97±8
β-Ionone	79-77-6	0.47-3.19	0.15	0.19	4.75	114±7
Terpenes						
Geraniol	106-24-1	7.50-50.21	0.43	0.57	2.31	97±5
Citronellol	106-22-9	4.52-30.28	0.90	1.73	4.91	101±4
Farnesol	4602-84-0	2.17-21.94	1.07	1.22	6.31	92±7
Linalool	78-70-6	6.34-42.09	1.14	2.00	1.87	113±13
Nerolidol	7212-44-4	1.88-12.50	0.67	0.96	5.70	91±6
Volatile phenols						
Guaiacol	90-05-1	12.53-82.15	2.68	6.76	5.00	108±9
Eugenol	97-53-0	9.48-62.80	6.60	6.62	2.09	114±9
Vanillin	121-33-5	1.03-30.32	0.09	0.21	1.82	115±12

LOD: instrument detection limit; LOQ: instrument quantification limit.
%RSD: percentage of relative standard deviation.
[Δ]Samples were diluted when values were out of linear range.
[†]Recovery values were calculated for an intermediate concentration in a model solution of synthetic wine.

Table S2. Linear range, LOD, LOQ, %RSD and recovery for phenolic compounds.

	CAS Nº	Linear range$^{\Delta}$ (mg/L)	LOD (mg/L)	LOQ (mg/L)	%RSD	%Recovery†
Flavanols						
(+)-Catechin	154-23-4	7.94-95.26	1.19	3.59	0.60	100±1
(-)-Epicatechin	490-46-0	18.38-219.89	4.16	12.61	1.66	100±2
Procyanidin B2	29106-49-8	0.46-23.10	0.35	1.05	0.21	99±2
Phenolic acids						
Ellagic acid	476-66-4	21.63-259.54	12.56	35.47	0.77	99±1
Gallic acid	149-91-7	3.98-47.76	0.71	2.15	0.33	100±3
Protocatechuic acid	99-50-3	1.11-13.27	0.02	0.05	0.77	100±1
Syringic acid	530-57-4	1.86-22.34	0.32	0.98	0.27	100±1
t-Caffeic acid	331-39-5	3.29-39.51	0.28	0.75	1.06	100±2
t-Coumaric acid	501-98-4	1.00-12.67	0.15	0.45	1.20	100±1
4-Hidroxybenzoic acid	99-96-7	10.26-123.08	0.11	0.32	0.25	100±2
Vanillic acid	121-34-6	2.08-24.91	0.18	0.59	1.02	100±1
Stilbenes						
t-Resveratrol	501-36-0	1.09-13.07	0.09	0.26	0.12	100±2
Anthocyanins						
Malvidin 3-O-glucoside	7228-78-6	9.52-247.07	2.54	4.14	0.50	99±1
Flavonols						
Quercetin 3-O-glucoside	482-35-9	1.02-12.23	0.56	0.82	1.69	99±2

LOD : instrument detection limit; LOQ: instrument quantification limit.
%RSD: percentage of relative standard deviation.
$^{\Delta}$ *Samples were diluted when values were out of linear range.*
†*Recovery values were calculated for an intermediate concentration in a model solution of synthetic wine.*

Table S3. Weights of the total variables (volatile and phenolic compounds, and sensorial descriptors) in the first two principal components.

		Component 1	Component 2
Sensorial descriptors	Purple	0.0688	0.0915
	Garnet	-0.0764	0.0481
	Red	0.0291	-0.0881
	Red fruits(O)	0.1151	-0.0277
	Nuts(O)	-0.0629	0.1680
	Herbaceous(O)	-0.0393	-0.1624
	Vanilla(O)	-0.0620	0.1723
	Toasted(O)	-0.0605	0.1754
	SEGs(O)	-0.0676	0.1618
	Red fruits(T)	0.0636	-0.0037
	Nuts(T)	-0.0648	**0.1812**
	Herbaceous(T)	0.0285	-0.1465
	Vanilla(T)	-0.0365	**0.1834**
	Toasted(T)	-0.0540	0.1753
	SEGs(T)	-0.0713	**0.1830**
	Dryness	-0.0966	-0.0587
	Silkiness	-0.0136	**0.2211**
	Bitterness	-0.0607	-0.1229
Volatile compounds	Ethyl decanoate	0.0205	-0.1725
	Ethyl acetate	0.0604	-0.0777
	2-Phenylethanol	0.1465	-0.0067
	Nonanol	0.1493	0.0266
	Ethyl cinnamate	0.1431	0.0893
	2-Phenyl ethyl acetate	**0.1584**	0.0138
	β-Ionol	0.0382	**-0.2065**
	β-Ionone	0.1088	0.1636
	Geraniol	0.1377	0.0238
	β-Ionone	0.1143	0.0130
	Citronellol	0.1388	-0.0470
	Farnesol	0.1386	-0.0276
	Linalool	**0.1581**	0.0147
	Nonanal	0.1067	-0.0074
	Ehyl lactate	0.1075	0.0044
	Ethyl octanoate	-0.0965	0.0687
	Ethyl butyrate	0.0696	-0.0828
	Diethyl succinate	0.1347	-0.0268
	Ethly hexanoate	0.1468	0.0924
	Isoamyl acetate	0.1166	-0.0098
	Benzyl alcohol	0.1397	0.0671
	β-Damascenone	**0.1585**	0.0403
	Hexyl acetate	0.1326	0.0845
	1-Hexanol	0.1337	0.0771
	3-Hexen-1-ol	0.1456	0.0744
	Hexanoic acid	0.1283	0.0713
	Octanoic Acid	0.1495	0.0033
	Decanoic acid	0.0711	-0.1387
	Ethyl vanillate	0.1440	0.0226
	Nerolidol	0.1367	-0.0823
	Eugenol	0.1343	0.0839
	Vanillin	0.1432	0.0496
	Guaiacol	0.0872	0.0919
	Benzaldehyde	0.0297	0.0650
Phenolic compounds	(+)-Catechin	0.0694	-0.1580
	(-)-Epicatechin	0.0609	-0.0949
	Procyanidin B2	-0.0847	-0.0490
	Ellagic acid	0.1294	-0.1086
	Gallic acid	-0.0579	**-0.1864**
	4-Hidroxybenzoic acid	0.0449	0.1297
	Protocatechuic acid	-0.0805	-0.1158
	Syringic acid	0.0924	0.1109
	t-Caffeic acid	0.0358	-0.0162
	t-Caftaric	-0.1019	-0.1560
	t-Coutaric acid	-0.0563	**-0.1918**
	Coumaric acid	-0.1196	0.1235
	Vanillic acid	0.0499	0.0279
	t-Resveratrol	-0.0836	**0.2034**
	Delphinidin 3-O-glucoside	**-0.1547**	-0.0166
	Cyanidin 3-O-glucoside	-0.1496	0.0324
	Petunidin 3-O-glucoside	**-0.1592**	-0.0005
	Peonidin 3-O-glucoside	-0.1491	0.0345
	Malvidin 3-O-glucoside	**-0.1594**	0.0418
	Malvidin 3-(6'-acetyl)-glucoside	-0.1492	0.0689
	Malvidin 3-(6'-*t*-caffeoyl)-glucoside	-0.1472	-0.0482
	Petunidin 3-(6'-*p*-coumaroyl)-glucoside	-0.1485	-0.0404
	Cyanidin 3-(6'-*p*-coumaroyl)-glucoside	-0.1490	0.0263
	Malvidin 3-(6'-*p*-coumaroyl)-glucoside	-0.1498	-0.0338
	Myricetin 3-O-glucurunide + Myricetin 3-O-glucoside	-0.1223	-0.0998
	Syringetin 3-O-glucoside	0.0014	**-0.1934**
	Myricetin	0.0107	**-0.2234**
	Quercetin	0.0226	**-0.2136**
	Myricetin 3-O-galactoside	-0.1479	0.0147
	Myricetin 3-O-glucuronide a	-0.1459	0.0072
	Laricitrin 3-O-glucoside/galactoside	-0.1477	0.0175

(O): descriptors of olfactory phanse; (T): descriptros of taste phase.
Compounds or sensorial descriptors with a weight higher than 0.15 in component 1 and 0.18 in component 2 typed in bold.

Figure S1. Linear regression for the differences respect to the control wine, associated with the four vine-shoots treatments during winemaking, for the descriptors related to the *SEGs* impact. a) Nuts; b) Vanilla; c) Toasted; d) SEGs; d) Silkiness tannin.

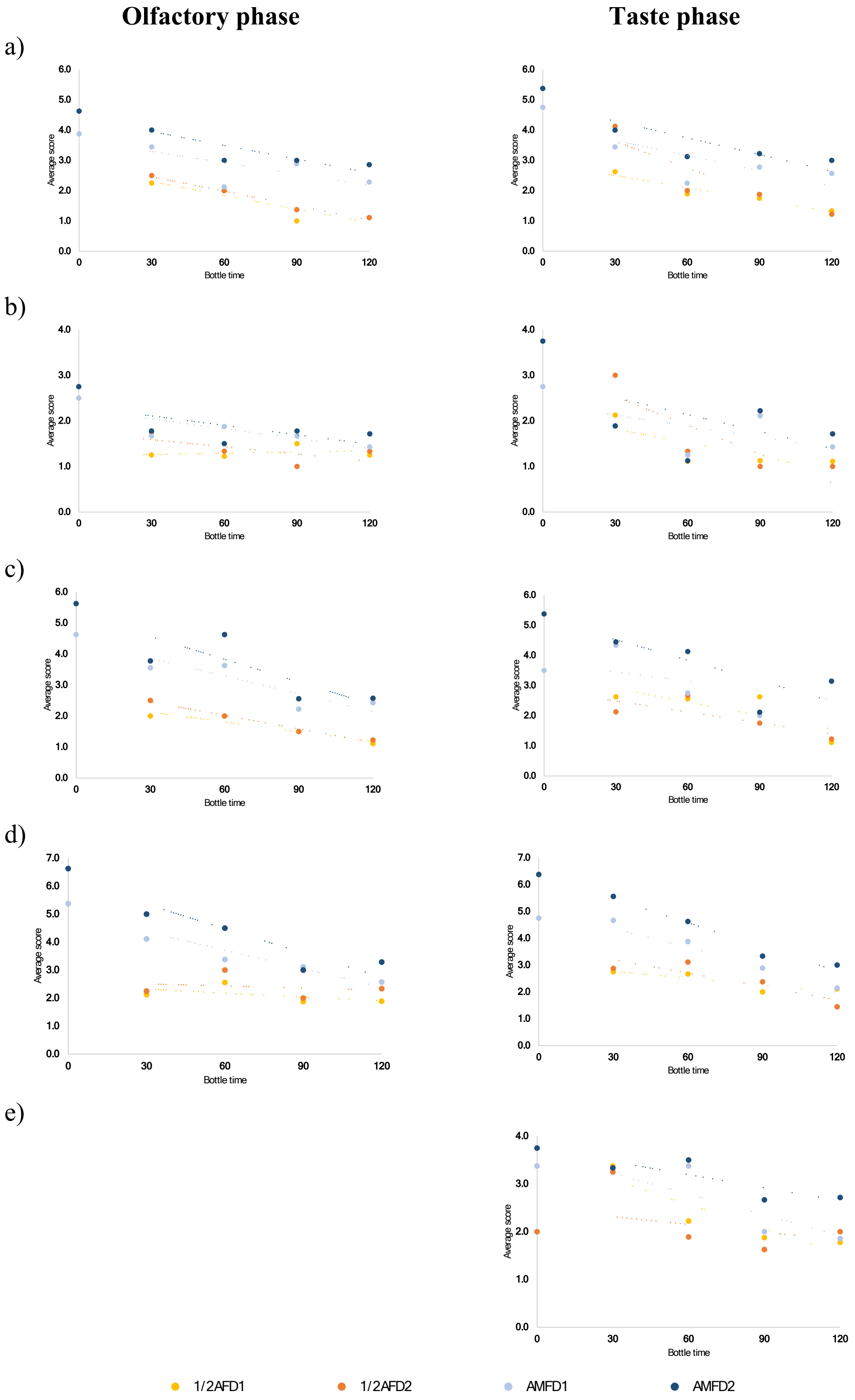

1/2AFD1: 12 g/L of SEGs added in middle of alcoholic fermentation; 1/2AFD2:24 g/L of SEGs added in middle of alcoholic fermentation; AMFD1: 12 g/L of SEGs were added in after malolactic fermentation; AMFD2: 24 g/L of SEGs were added in after malolactic fermentation.

26
